# Photoperiodic patterns in miRNA-mRNA pairs and tRNA fragments revealed by time-course co-sequencing in Arabidopsis

**DOI:** 10.1371/journal.pgen.1012229

**Published:** 2026-07-22

**Authors:** Chun Chung Leung, Daniel A. Tarté, Joshua M. Gendron

**Affiliations:** Department of Molecular, Cellular and Developmental Biology, Yale University, New Haven, Connecticut, United States of America; CNRS UMR8120: Genetique Quantitative et Evolution Le Moulon, FRANCE

## Abstract

In plants, the response to photoperiod is marked by global reprogramming of gene expression that drives extensive developmental changes. Small RNAs (sRNAs) play important roles in this process, but a comprehensive characterization of micro RNAs (miRNAs) and the more recently recognized transfer RNA fragments (tRFs) within this context is lacking. Herein, we characterize the patterns of miRNAs and tRFs in Arabidopsis by performing time-course sRNA-sequencing across three photoperiods. By comparing with our previous messenger RNA (mRNA) sequencing time-courses, we identified positively- or inversely-correlated miRNA-mRNA pairs between the two co-sequenced datasets that suggest photoperiodic sRNA regulations. Furthermore, we revealed 20 patterns of photoperiodic tRFs. These patterns are linked to the transfer RNA (tRNA) isotypes and positions they derive from, suggesting that tRFs are subjected to photoperiodic regulation. Finally, we present a major update to our web-app “Photo-Graph,” (http://gendron-lab.shinyapps.io/PhotoGraph) featuring new visualizations of this mRNA-sRNA co-sequencing dataset. In summary, our findings indicate that plants regulate sRNAs within a diel cycle in a photoperiodic manner and form highly-correlated pairs with mRNAs.

## Author summary

Plants regulate gene expression in response to changes in day length, *i.e.,* photoperiod, in order to coordinate growth and development. In previous studies, we showed that the precise pattern of gene expression in a 24-hour day is tightly linked to photoperiodic biological processes. Herein, we used time-course co-sequencing to study how patterns of two types of small RNAs — micro RNAs (miRNAs) and transfer RNA fragments (tRFs) — are correlated to patterns of mRNAs. miRNAs have long been known as regulators of photoperiodic flowering. We revealed several miRNAs that show high correlation with target mRNAs. Notably, miR398 and the target genes involved in oxidative stress show concordant diel patterns across three photoperiods. Compared to miRNAs, tRFs are a more newly recognized small RNA type. In addition to showing that the levels of specific tRFs display characteristic diel patterns, we also demonstrated that the loci that these tRFs derived from are also correlated to photoperiod.

## Introduction

Plants sense and respond to day length - or photoperiod - in order to coordinate developmental and reproductive processes in anticipation of seasonal environment changes [1,2]. This coordination manifests in the myriad of light signaling pathways, circadian clock mechanisms and gene regulation that convert photoperiodic information into physiological outputs including flowering [3], hypocotyl elongation [4] and growth rate adjustment [5]. A preeminent model for photoperiodism (response to photoperiod) is the regulation of Arabidopsis floral transition. Flowering in *Arabidopsis thaliana* (Arabidopsis) is controlled by a mechanism involving CONSTANS (CO) and its target gene *FLOWERING LOCUS T* (*FT*), where a sufficiently long daytime causes light-activated stabilization of CO protein in the later part of a day, and induces expression of the florigen-encoding *FT* gene [6]. This mechanism ensures flowering at a time optimal for reproductive fitness. Photoperiodism is also observable in a non-reproductive context. A short-day photoperiod increases nighttime hypocotyl elongation rate in Arabidopsis seedlings through a PHYTOCHROME-INTERACTING FACTOR (PIF)-dependent process, in which the dark-stabilization of the PIF proteins can only take place after a sufficiently long period of darkness [7,8]. The CO-FT and the PIF modules share a mechanistic similarity where a response is triggered upon the coincidence between an external light condition and an internal protein level rhythm — this phenomenon is described by the external coincidence model in photoperiodism.

A photoperiodic mechanism separate from the CO-FT and PIF modules is metabolic day length measurement (MDLM) [5,9]. MDLM controls the mid-day peaking of gene expression in darkness under a short photoperiod, or in the light under a long photoperiod. For instance, the MDLM-regulated senescence gene *PHLOEM PROTEIN 2-A13* (*PP2-A13*) displays a short-day-specific peak in the dark at Zeitgeber Time 12 hour (ZT12) [9], while *MYO-INOSITOL 3-PHOSPHATE SYNTHASE 1* (*MIPS1*) shows a long-day-specific peak in the light at ZT12 [10]. An important observation about MDLM-regulated genes is that the direction of daily expression integral (DEI), *i.e.,* the sum of expression over a 24-hour day, may indicate the photoperiod under which the gene’s function is more important. This is the case for the short-day-induced *PP2-A13* and the long-day-induced *MIPS1*, for which the loss-of-function mutation causes a short-day-specific and long-day-specific phenotype, respectively [9,10].

The identification of photoperiodically expressed genes is pivotal to the elucidation of day length measurement mechanisms at a molecular level. Using microarray time-courses in various photoperiods and light conditions, the DIURNAL project identified *cis*-elements associated with different phases of gene expression [11,12]. In two previous studies by our group, microarray and RNA-sequencing time courses were separately analyzed with the relative DEI (rDEI) metric to identify photoperiodically induced genes [9,13]. With a focus on rDEI - the relative sum of expression between short-day and long-day, or between any two photoperiods - our group identified approximately 2000 genes that are likely regulated by the MDLM system. Moreover, we also highlighted the existence of at least 14 other photoperiodic gene expression patterns that are correlated to specific physiological processes and *cis*-elements.

microRNAs (miRNAs) are important regulators of gene expression. A class of 20–24 nucleotide (nt) long small RNAs (sRNAs) — miRNAs mediates gene expression silencing through the targeting of cognate transcripts by complementary base pairing, followed by direct mRNA cleavage or translation inhibition [14,15]. In the case of gene silencing by cleavage, there are at least two possible modes of action: A) a clearance mode where the mRNAs are actively cleaved and miRNA-mRNA pairs show opposite expression patterns, and B) a rheostat-like buffer mode where miRNAs and cognate mRNAs are co-expressed, and miRNAs act to dampen noise in mRNA expression [16,17]. Within the clearance mode an inverse relationship may exist between the sRNA-mRNA pair. This can be observed among several miRNA-mRNA pairs in Arabidopsis, *e.g.,* the inverse gradient between miR166s and *PHABULOSA* that determines xylem cell specification in roots [18,19], and that between miR156s and transcripts encoding SQUAMOSA PROMOTER BINDING PROTEIN-LIKE (SPL) family transcription factors in the control of vegetative phase transition [20]. On the other hand, positive correlations in miRNA-mRNA pairs have also been reported in many cases. A comprehensive survey in *Oryza sativa* found positive instead of negative correlations in 22 out of 51 significantly correlated miRNA-mRNA pairs [21]. In Arabidopsis, miR395 and the target *SULFATE TRANSPORTER 1;2* (*SULTR1;2*) display positive correlation temporally across a two-week-long sulfur starvation treatment, but negative correlation can be seen across leaf and root tissues [22]. Positively correlated miRNA-mRNA pairs may represent a rheostat-like buffering mechanism for expression stabilization, feedback loops [23], or potentially other gene expression mechanisms.

miRNA pathways interact with photoperiodic flowering. In long day conditions, miR172 down-regulates the direct *FT*-repressor *SCHLAFMUTZE* (*SMZ*), as well as other flowering repressors, e.g., *SNARCHZAPFEN* (*SNZ*) and *TARGET OF EARLY ACTIVATION TAGGED 1* (*TOE1*); this promotes *FT* expression and thus flowering [24]. miR172 level itself is regulated in a photoperiodic manner dependent on the circadian clock component GIGANTEA [25]. Apart from miR172, several miRNAs also control flowering time through non-photoperiodic mechanisms. For instance, the miR399-*PHOSPHATE 2* module controls temperature-regulated flowering [26] and the miR393 module controls auxin-mediated flowering [27].

In addition to miRNAs, tRNA fragments (tRFs) also represent a class of sRNAs important for gene regulation. Although tRFs were initially thought to represent only byproducts of tRNA degradation, recent findings, however, show that tRF levels respond to biological stresses and regulate gene silencing [28]. Currently, tRFs are generally categorized into two types based on their sizes: 1) larger 30–40 nt long tRF-halves that are derived from cleavage at the anticodon loop, and 2) smaller 17–26 nt long tRFs formed from cleavage around the D loop (5′ tRFs), from cleavage around the T loop (3' tRFs), and those produced from the 3' end of tRNA precursors (3' U-tRFs) [28,29]. tRF processing shares similarity with that of miRNAs. A study of Arabidopsis pollen and inflorescence tissues reveals that 19 nt 5′ tRFs derived from tRNA-Ala^*AGC*^ (5′ tRF-Ala^*AGC*^) are processed by Dicer-like 1 (DCL1) and associate with Argonaute1 (AGO1) [30]. The same study also demonstrated that tRFs may mediate target transcript cleavage in a manner akin to miRNA. 5′ tRF^*CAT*^ was shown to initiate the cleavage of the transposable element transcript *Athila6a* by a DCL1-dependent and partially AGO1-dependent mechanism [30]. Another study in Arabidopsis seedlings showed that tRFs have a role in inhibiting global protein translation in the FERONIA-YUELAO (FER-YL) pathway [31]. Through the control of tRNA methylation, FER and YL regulate the abundance of a subgroup of 5′ tRFs and in turn control root hair growth phenotypes. Furthermore, the levels of specific tRFs have been associated with various abiotic and biotic stresses. For instance, 5′ tRF-Arg^*TCG*^ and 3'tRF-Tyr^*GTA*^ are induced under oxidative treatment with hydrogen peroxide in Arabidopsis [29], while 5′ tRF-Ala^*AGC*^ are induced under fungal infection and regulate anti-fungal defense [32].

Systematic profiling of diel gene expression patterns has proven a powerful approach to identify photoperiodic mechanisms. Our previous studies showcased that photoperiodic gene expression patterns in a diel timescale may generate photoperiodic growth responses observable in a developmental timescale [9,10,13]; we herein exploit a co-sequencing approach and perform time-course sRNA-sequencing (sRNA-seq) on the same samples in our previous mRNA-seq study [13] to investigate the diel photoperiodic levels of sRNAs. We focus our library construction and analyses on miRNAs and tRFs: the miRNA miR172 is a well-characterized regulator of photoperiodic flowering [33], but it is less clear whether miRNA levels in general show photoperiodic regulation within a single day; on the other hand, although tRFs have not been directly linked to photoperiodism, levels of specific tRFs have been correlated with many responses to environmental stresses, *e.g.,* drought, UV light and cold stresses [34], which are expected to vary with seasonal timings. This work exploits the co-sequencing technique to identify miRNA-mRNA and tRF-mRNA pairs, in order to provide insights in how diel photoperiodic expression patterns may associated with both known and unknown photoperiodic biological processes.

## Results

### A mRNA-sRNA co-sequencing dataset across three photoperiods is designed to study gene expression in a diurnal time scale

**Fig 1:**
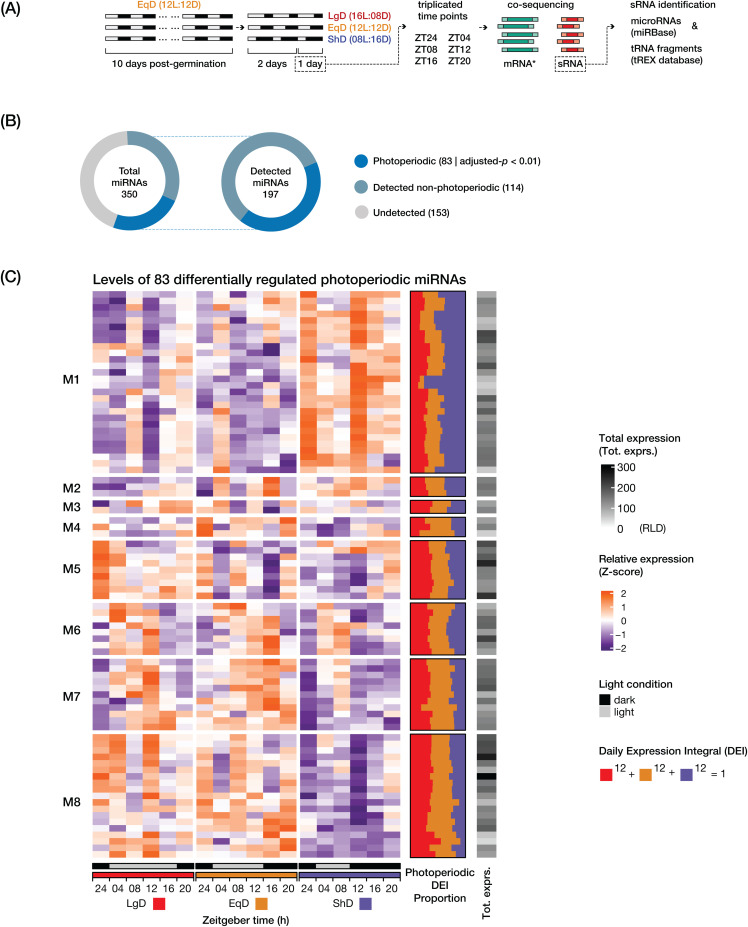
Time-course sequencing reveals sRNAs that show photoperiodic diurnal patterns. **A** The design of the sampling scheme and co-sequencing datasets. The bulk mRNA-sequencing dataset has been published previously [13]. Gray and dark bars represent light and dark periods, respectively. Zeitgeber time is set against dawn (*i.e.,* ZT00). The first sampling time point is ZT24, *i.e.,* in darkness just before dawn (see Materials and methods). **B** Pie charts illustrating the number of detected photoperiodic miRNAs. **C** Main body: heat map shows the clustering of 83 photoperiodic miRNAs into eight groups (M1 - M8; likelihood ratio test: adjusted-*p* < 0.01); Photoperiodic DEI Proportion: stacked bar chart of the proportional daily expression integral (DEI) of each gene, raised to the power of 12 for visualization (red = LgD; orange = EqD; blue = ShD); total expression: heat map of the total expression in RLD-normalized read counts. Names of selected miRNAs are labeled on the right.

To investigate the roles of sRNAs in photoperiodism in a daily time scale, we designed a sRNA-co-sequencing dataset paired with our previous mRNA-sequencing time courses (Fig 1A) [13]. Given the s/mRNA libraries were generated from the same total RNA samples, and that sRNAs generally regulate biological processes via targeting mRNAs, we exploit this co-sequencing strategy and consider a sRNA-mRNA pair with highly correlated expression patterns between photoperiods to be candidate sRNA-mediated regulations. Furthermore, this dataset also shares the advantages of the previous mRNA-sequencing time courses: **1)** coverage of three photoperiods, *i.e.,* 16-hour long day (LgD; 16 hours of light followed by 8 hours of darkness; 16L:08D), 12-hour equinox day (EqD; 12L:12D) and 8-hour short day (ShD; 08L:12D); **2)** the developmental timing of 13 days post-germination, which is late enough for the perception of the long-day flowering signal but earlier than the morphological floral transition; **3)** 2 days of photoperiod entrainment after 10 days of EqD-growth, which is a method to synchronize growth stage without triggering an observable stress response [13]; **4)** six time points with biological triplication for statistical power.

Reads were assigned to unique sequences of known mature miRNAs and tRFs documented on miRBase [35] and the tREX database [36], separately (Materials and Methods; S1 Data). We examine these two sRNA types rather than other larger structural RNAs, *e.g.,* small nucleolar RNAs and ribosomal RNAs, to focus on photoperiodic mRNA transcript regulations.

### ShD- and LgD-specific double-peaked patterns are observed in miRNAs

sRNA-sequencing reveals that 42% of the detected miRNA sequences (83 out of 197; adjusted-*p* < 0.01) show differential levels across photoperiods (Fig 1B and S2 Data). Clustering analysis groups miRNAs into eight groups (M1 - M8) (Fig 1C and S3 Data). The miRNA groups M1 shows overall high ShD level, with peaks at ShD-ZT24, *i.e.,* at the end of nighttime, and ShD-ZT12, *i.e.,* first time point after dusk at ShD-ZT08. This pattern is highly reminiscent of the double-peaked pattern of ShD-induced genes controlled by the metabolic day length measurement (MDLM) mechanism [9,13]. Induction towards longer photoperiods, *i.e.,* LgD and EqD can be observed, in group M5/6/7/8. M5 displays pronounced peaking at LgD-ZT24 (end of nighttime), but no observable peaking in EqD or ShD. M8 generally shows high levels in LgD-ZT24/08/12, and EqD-ZT16/20, but patterns are varied. Patterns of M6/7 are also varied, but largely exhibit high expression levels at LgD-ZT12 and EqD-ZT16.

We also compared miRNA expressions with bulk mRNA-sequencing time courses. Under the same statistical criteria, differential expression is observed in 76% of detected mRNAs (15936 out of 21003; adjusted-*p* < 0.01) (S1 and S2 Figs and S4 and S5 Data). This aligns with previous studies which report the majority of transcripts cycle with time of day [12]. ShD-specific double-peaked patterns that are signature of MDLM can be observed in C13 (428 mRNA) and C19 (1538 mRNAs), while the MDLM-signature LgD-specific double-peaked patterns in C2 (2041 mRNAs) (S2 Fig). Overall, this shows that the MDLM-signature diel photoperiodic patterns can be observed in both miRNAs and mRNAs. Furthermore, end-of-night and dusk are two time points where a substantial proportion miRNAs and mRNAs show photoperiodic changes.

### Co-sequencing approach reveals positively- and negatively-correlated miRNA-mRNA pairs

To investigate the miRNA-mRNA relationships between photoperiods, we exploited the co-sequencing approach and calculated correlations between miRNAs and their literature-reported mRNA targets in three databases: miRTarBase [37], TarDB [38] and PmiREN [39]. A total of 470 unique pairs are reported by these databases (S6 Data). Highly correlated miRNA-mRNA pairs, whether positively or negatively correlated, may indicate potential *bona fide* miRNA-regulation, given that both RNAs coexist at detectable levels.

First, we asked if a general trend towards positive or negative correlation can be seen in the miRNA-mRNA pairs. Of the 470 unique pairs, 99 show differential levels in both miRNA and mRNA. Within these 99 pairs, 15 pairs show negative correlation (Pearson’s Correlation Coefficient ρ <-0.3) while 10 show positive correlation (ρ > 0.3) across all samples (S3 Fig and S6 Data). To examine if the miRNA-mRNA pairs would be more strongly correlated in a specific photoperiod, which would be indicative of a photoperiodic effect on all miRNA-mRNA regulations, we also calculated correlation using only LgD, EqD or ShD samples (S3 Fig and S6 Data). In LgD-, EqD and ShD-only comparisons, 19 versus 30 pairs, 15 versus 11, 22 versus 14 show negative correlation and positive correlation, respectively. Overall, LgD photoperiod shows slightly more positively correlated pairs compared to other photoperiods. To further examine if this change in global pattern in miRNA-mRNA correlations is statistically significant, we used Quantile-Quantile plots (Q-Q plots) to compare the observed *p*-values of the Pearson’s correlation tests against an expected distribution (S4 Fig). We noticed that statistically significant correlations, regardless of direction, are more numerous in LgD and ShD photoperiods, compared to EqD. However, we did not identify a substantial bias towards positive or negative correlations in miRNA-mRNA pairs in a specific photoperiod.

Next, we asked if miRNA-mRNA correlations may exist across time points. This study used 4-hour time courses, and it is possible that a change in miRNA level only causes a change in mRNA steady state level in the next time point. To test this, we shifted the data of mRNA levels forward by one time point. The mRNA ZT24 time point was paired with miRNA ZT20 time point due to the cyclical nature of the time courses, and the correlation and Q-Q plot analyses were performed between the miRNA levels and the log2-fold-change of mRNA expression (S5 and S6 Figs and S7 Data). Overall, we observed slightly lower number of miRNA-mRNA pairs with statistically significant negative or positive correlations, suggesting that miRNA-mRNA regulations across 4-hour time points are likely relatively rare. Nevertheless, we detected several negatively correlated pairs, including miR396b-*ACHT5* (*ATIPICAL CYS-HIS-RICH THIOREDOXIN 5*), miR170-*HAM3* (*HAIRY MERISTEM 3*) and miR398a-*AT5G14550*, which may represent possible miRNA-regulatory pairs (S7 Fig).

#### Patterns of miR398, miR775 and miR400 are highly correlated with target mRNAs.

**Fig 2:**
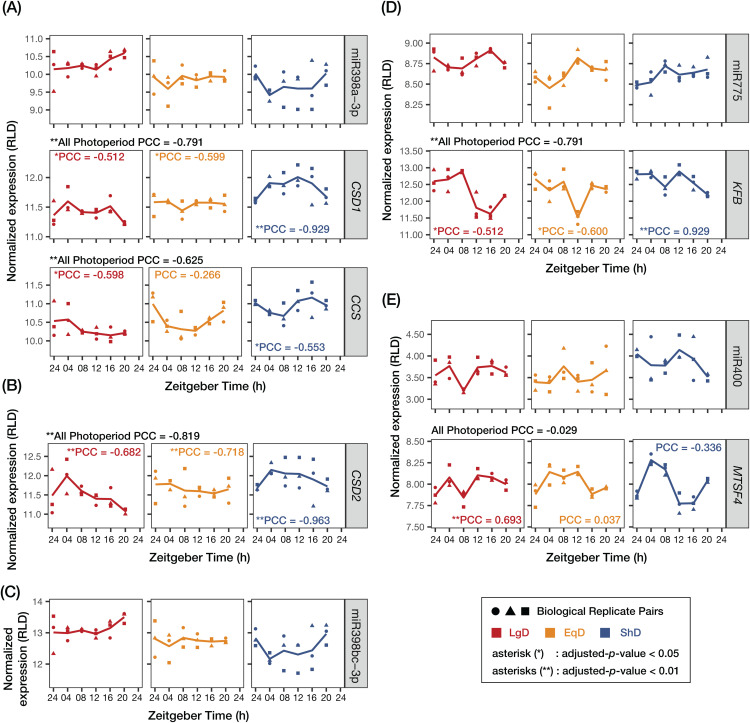
Negative and positive correlations are observed in reported miRNA-mRNA pairs. Expression levels of selected miRNAs and mRNAs. Lines indicate mean and colors indicate photoperiod (red = LgD; orange = EqD; blue = ShD). Data points are represented in squares, triangles or circles; the shapes represent the pairing of total RNA samples between s/mRNA-co-sequencing. Asterisk(s) (*) indicates statistical significance of the test of Pearson’s correlation coefficient (PCC). Single (*) and double (**) asterisks indicate adjusted-*p* < 0.05 and ** adjusted-*p* < 0.01, respectively. Expressions are in regularized-log-transformed values (RLD). **A** Expression levels of miR398a, *CSD1* (AT1G08830) and *CCS* (AT1G12520). **B** Expression levels of *CSD2* (*AT2G28190*). **C** Expression level of miR398bc. **D** Expression levels of miR775 and *KFB* (*AT1G23390*). **E** Expression levels of miR400, and *MTSF4* (*AT4G19940*).

In order to understand what biological processes might be regulated by the photoperiodic miRNA-mRNA levels, we further examined the patterns of individual miRNA-mRNA pairs. Of the 99 pairs where both RNAs are differentially regulated, 32 show statistically significant correlations either within one photoperiod or across all three (Pearson’s correlation test; adjusted-*p*-value < 0.05; Fig 2A and S6 Data). Out of these 32 pairs, 14 were positively correlated and 18 were negative. Below, we highlight three miRNAs, miR398, miR775 and miR400, and their highly correlated targets.

We observe high negative correlation between miR398a and the targets *COPPER/ZINC SUPEROXIDE DISMUTASE 1* (*CSD1*) and *COPPER CHAPERONE FOR SOD1* (*CCS*), which is especially strong in ShD (Pearson’s correlation coefficient PCC = -0.929 for miR398a-*CSD1* and PCC = -0.553 for miR398a-*CCS*; both at adjusted-*p* < 0.01; Fig 2A and S6 Data). Interestingly, the miR398a level is highest in LgD and lowest in ShD, while the inverse pattern is seen in *CSD1* and *CCS*. The miR398-CSD (COPPER/ZINC SUPEROXIDE DISMUTASE) module regulates the scavenging of reactive oxygen species (ROS) in plant cells [40,41]. Stressed-induced ROS repress the levels of miR398s, and thus in turn induces CSD1/2 and COPPER CHAPERONE FOR SOD1 (CCS). This upregulation of CSD1/2 and CCS reinforces ROS scavenging in plant cells. Although the miR398a-*CSD2* was not tested initially because the *CSD2* mRNA was not considered differentially expressed (adjusted-*p*-value ≈ 0.0185), we notice that the *CSD2* pattern also negatively correlates with miR398a (PCC = -0.963 in ShD, PCC = -0.819 in all photoperiods; Fig 2B). Furthermore, miR398bc, which was also classified as non-differentially expressed across photoperiods (adjusted-*p*-value ≈ 0.011), shows a similar pattern with miR398a (**Fig 2C**). Overall, the miR398-module as a whole shows congruent negative correlations across photoperiods, and a trend towards higher mRNA levels in shorter photoperiods.

miR775 is a regulator of root growth and leaf size [42,43], and is a direct target of the transcription factor LONG HYPOCOTYL 5 (HY5), a growth regulator rapidly degraded in a light-to-dark transition [42,44]. In this study, we observe that miR775 level peaks at the pre-dusk time point in each photoperiod, *i.e.,* LgD-ZT16, EqD-ZT12 and ShD-ZT08 (Fig 2D); this aligns with the known rhythmic level of HY5 protein [44]. The expression of *KELCH DOMAIN-CONTAINING F-BOX PROTEIN* (*KFB*; *AT1G23390*), a predicted miR775 target [37], and a likely candidate of HY5 regulation, shows a highly inversely correlated pattern, where a marked drop at the same pre-dusk time points can be observed. *KFB* encodes a Kelch domain-containing F-box protein that controls flavonoid biosynthesis, and it strongly responds to light-to-dark and dark-to-light switches [45]. Overall, the miR775-*KFB* pair shows strong negative correlation.

We observed significant positive correlation in the pair miR400-*MTSF4* (*MITOCHONDRIAL STABILITY FACTOR 4*; Fig 2E; PCC = 0.693 in LgD; Fig 2E) [38]. Both miR400 and *MTSF4* show sharp drop at ZT08 specifically in LgD and are not strongly correlated in EqD and ShD. In addition to the roles in plant defense [46] and ROS regulation [47], miR400 is also an anterograde regulator of *MTSF4* in heat stress response [48]. *MTSF4* encodes an essential pentatricopeptide protein that regulates mitochondrial transcript levels [48]. Knockout of *mtsf4* is embryonic lethal, and low level of *MTSF4* also causes growth defects in later developmental stages. Intriguingly, the LgD-specific dip of both miR400 and *MTSF4* indicates positive correlation, but previous report by Jung et al (2023) showed negative regulation under heat stress, suggesting the miR400-*MTSF* regulation may be dependent on environmental context.

In addition to miR398, miR775 and miR400, we also identified highly correlated pairs for miR156i, miR156j, miR164c, miR393ab, miR396b and miR8182 (S8 Fig). Although these pairs are well supported by the respective studies and databases [37,38,49], their biological functions and implications are physiology are considerably less well understood. Taken together, while the majority of the known miRNA-mRNA pairs are not correlated in a photoperiod-specific manner within a diel time scale, several miRNAs, *e.g.* miR775, miR400 and especially miR398a shows strong correlation with the downstream targets, suggesting diel patterns of miRNA-mRNA regulations play a role in photoperiodism.

### Multiple modes of ShD-, LgD- and EqD-induction are observed in photoperiodic tRFs

**Fig 3:**
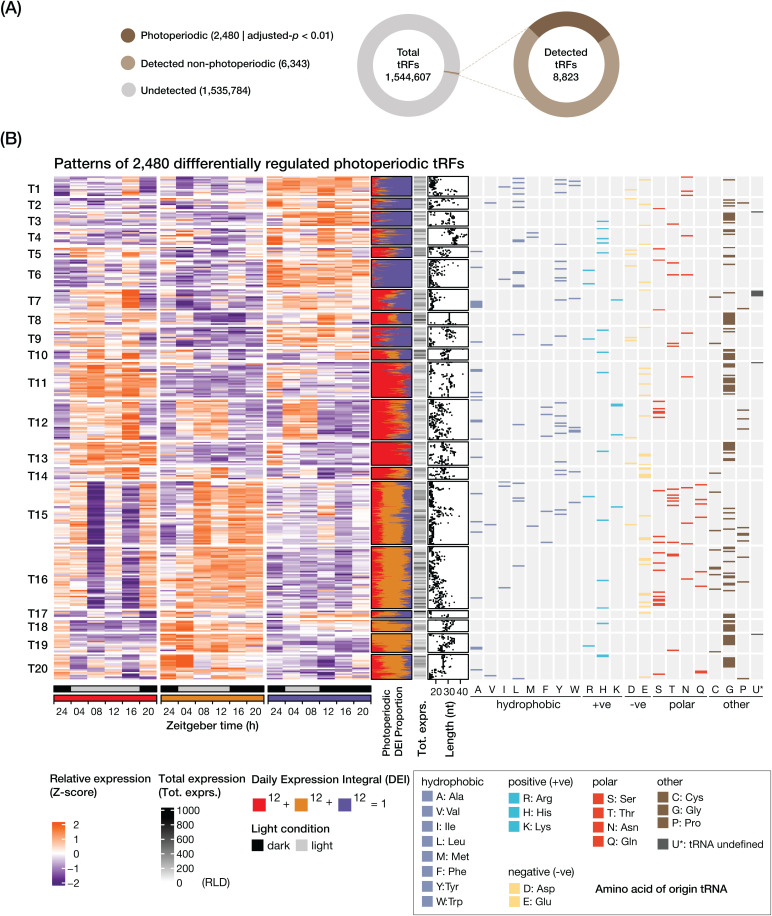
Time-course sequencing reveals that the photoperiodic patterns of tRFs in relation to fragment size and tRNA types. **A** Pie charts illustrating the number of detected photoperiodic tRFs. **B** Main body: heat map shows the clustering of 2,480 photoperiodic tRFs into twenty groups (T1 - T20; likelihood ratio test: adjusted-*p* < 0.01); Photoperiodic DEI Proportion: stacked bar chart of the proportional daily expression integral (DEI) of each gene, raised to the power of twelve for visualization (red = LgD; orange = EqD; blue = ShD); total expression: heat map of the total expression in RLD-normalized read counts. Length: dot plot showing the length of tRF at each row in number of nucleotides (nt). Amino acid of origin tRNA: twenty one heat maps showing the amino acid that the origin tRNA of the tRF carries. Amino acids are grouped by biochemical properties. U refers to tRNA-AT1G57710, for which the isotype is undetermined according to the tREX database [36].

**Fig 4:**
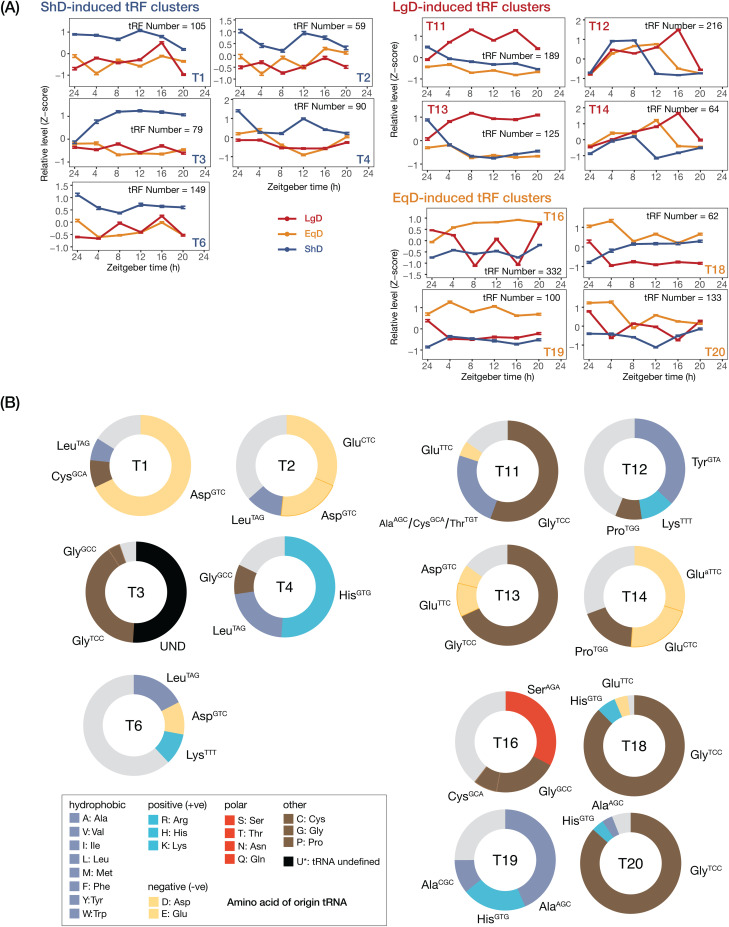
The top tRF types of tRF clusters. **A** Mean abundance level of tRFs in the clusters T1/2/3/4/5 (left), T10/11/12/13 (upper right) and T14/15/19/20 (lower right). For every tRF, the relative level is calculated by Z-score normalization. Error bars indicate standard error of the mean. The colors indicate photoperiod (red = LgD; orange = EqD; blue = ShD). tRF number: the number of unique tRF sequences in the cluster. **B** Pie charts showing the proportion of the top 3 abundant tRF types by read count (in reads per million, *i.e.,* RPM) for clusters T1/2/3/4/5 (left), T10/11/12/13 (upper right) and T14/15/19/20 (lower right).

A number of studies have shown how the abundance of tRFs may vary with various stresses. [28,34,50–53]. Stress, both biotic and abiotic, occur at different frequencies across yearly timings. Given plants exhibit photoperiodism to time biological processes to favorable seasons, we aimed to characterize the photoperiodic and diel regulations of tRFs; this may provide insights into potential photoperiod-controlled pathways that involve tRFs. To identify photoperiodic tRFs, we mapped sRNA reads to tREX database-annotated tRFs (Fig 3A and S8 and S9 Data). 28% of the detected tRF sequences are differentially regulated by photoperiod (2480 out of 8823; adjusted-*p* < 0.01, Fig 3A). Next, we categorized the tRFs into twenty clusters based on patterns (T1 - T20) (Figs 3B, 4A and S9–S29). Marked ShD-induction can be observed in T1/2/3/4/6. On the other hand, LgD-induction can be seen in T11/12/13/14. Of these clusters, the characteristic ShD-induced MDLM double-peaked pattern (peaking at ShD-ZT24 and ShD-ZT12 [9]) can be seen in T1/2/4/6, and the LgD-induced MDLM pattern (peaking at LgD-ZT16 [10]) in T12/14, suggesting that tRFs are potentially regulated by MDLM directly or indirectly. On the other hand, patterns different from the MDLM patterns are also observed. For instance, cluster T3 shows a ShD-induced pattern where ShD-level gradually increases from ShD-ZT24 throughout the day, and cluster T13 shows a similar pattern but for LgD-induction, where LgD-level is high except at LgD-ZT24. EqD-induced patterns, which have not been linked to the MDLM mechanism, can be seen in T16/18/19/20. T16/19 show high levels at EqD specifically at most time points across the time course, while T18/20 show high EqD-levels generally at EqD-ZT24/04.

### tRF patterns are correlated to size, tRNA of origin and reported biological responses

Compared to miRNAs, tRFs occurs in a larger size range (approximately 15 nt to 40 nt) and recent literature suggests their biological roles are correlated to the tRNAs they derive from [28,34]. Therefore, for each tRF we also annotated the fragment size, as well as the predicted origin tRNAs using the tRex database annotation (Figs 3B and S10–S29). We also identified the top 3 abundant tRF types by reads per million (RPM) in each cluster (S10–S29 Figs). Strikingly, we found that the clustering based solely on the abundance levels also separates tRFs by their size and origin tRNA, supporting that the photoperiodic tRF patterns are non-random and are linked to their biogenesis processes (Fig 3B). To succinctly present these patterns, we refer to tRF types by the tRNA regions they map to. *e.g.* tRFs that map to tRNA-Alanine-AGC are termed tRF-Ala^*AGC*^, and those that map equally stringently to both tRNA-Thr-TGT and tRNA-Gly-CCC are tRF-Thr^*TGT*^/Gly^*CCC*^.

#### ShD-induced long 5′ tRF-Asp^*GTC*^ are correlated to hypoxia stress.

We first inspected T1/2/3/4/6, which contain ShD-induced tRFs (Fig 4A and 4B). 5′ tRF-Asp^*GTC*^ is the most abundant tRF in T1 (S10 Fig), as well as the second most abundant in T2 (S11 Fig) and T6 (S15 Fig). Most of the 5′ tRF-Asp^*GTC*^ in cluster T1 are longer at 35 nt long (trex179959, trex253246 and trex1321415), spanning the 5′ half of the original tRNA. In contrast, those in T2 and T6 are shorter, at 26, 27 or 30 nt long in T2 and 19, 22 and 24 nt long in T6. Recent reports show that in Arabidopsis, 31 nt tRF-Asp^*GTC*^ can trigger the induction of immunity response against virus and aphids [54], and in human kidney cells, the accumulation of long tRF-Asp^*GTC*^-halves is associated with hypoxic stress [55]. It is notable that mRNA genes responsive to hypoxia are also found highly induced in ShD by MDLM [13]; this aligns with the marked peak at ShD-ZT12 of T1 where long 5′ tRF-Asp^*GTC*^ are found, which is indicative of ShD-MDLM regulation. Lastly, we also found an abundance of different types of tRF-Leu^*TAG*^, tRF-Glu^*CTC*^ and tRF-His^*GTG*^ in T1/2/4/6, which shows patterns similar to the characteristic ShD-MDLM pattern.

#### LgD-induced tRFs are related to phosphate starvation.

Next, we looked for the major tRF types that show LgD-induction in the clusters T11/12/13/14. In T11/13, 5′ tRF-Gly^*TCC*^ is the most abundant tRF type (Figs 4B, S19 and S22). However, notable size variation in the 5′ tRF-Gly^*TCC*^ is seen in the two clusters: T11 contains mostly 19 nt 5′ tRF-Gly^*TCC*^, while T13 mostly 26 nt 5′ tRF-Gly^*TCC*^. The production of twelve types of 19 nt-long 5′ tRF-Gly^*TCC*^ have been reported in Arabidopsis seedling roots subjected to phosphate starvation treatments [56]. Of these twelve, one of them (trex1082) are present in T11 (S9 Data). Therefore, it is reasonable to hypothesize that a biological process akin to the root phosphate-starvation response was occurring in LgD condition. Clusters T12/14, which exhibit a pattern resembling LgD-induced MDLM genes, show an abundance of 16 nt 5′tRF-Tyr^*GTA*^, 5′ tRF-Glu^*TTC*^, 5′ tRF-Glu^*CTC*^ and 3′ tRF-Pro^*TGG*^ (S21 and S23 Figs). These tRF types have not been reported in any study to our knowledge. Therefore, future experiments may be performed to test the biological functions of these tRFs.

#### EqD-induced tRFs are associated with various abiotic stresses.

The EqD-induced clusters T16, showing an overall high EqD level across all time points but EqD-ZT24, contains short 5′ tRF-Ser^*AGA*^, 3′ tRF-Gly^*GCC*^ and 3′ tRF-Cys^*GCA*^ below 29 nt (Figs 4A, 4B and **S25**). These tRF are not restricted to one size, but are mostly below 25 nt in length. On the other hand, cluster T19 with high EqD level across all time points contains 22–25 nt 5′ tRF-Ala^*AGC*^, 29-nt 5′ tRF-His^*GTG*^ and 23–25 nt 5′ tRF-Ala^*CGC*^. So far, these tRF types have not been characterized, but their high levels at many time points over EqD photoperiod suggest a potential important biological function.

Clusters T18/20 show highly similar EqD-induced patterns that peak at EqD-ZT24 and EqD-ZT04, with slight differences in their LgD and ShD patterns, and both T18 and T20 predominantly contain 5′ tRF-Gly^*TCC*^ (Figs 4A, 4B, S27 and S29). These EqD-induced 5′ tRF-Gly^*TCC*^ are of longer length than the 19 nt 5′ tRF-Gly^*TCC*^ in the LgD-induced cluster T11, indicating that they are generated from a different biological processes. Interestingly, T20 also contains an abundance of 19-nt-long 5′ tRF-Ala^*AGC*^, a type of tRF that has been shown to be induced under cold, drought and salt stresses [52]; these stresses are not expected to be more common in seasons with EqD photoperiod, suggesting that the photoperiodic diel patterns of these tRFs are not connected to those stress responses. Among the 19-nt-long 5′ tRF-Ala^*AGC*^ present in T20, trex516 (GGGGATGTAGCTCAGATGG), is known to negatively regulate *CYTOCHROME P450 71A13* (*CYP71A13*) expression and camalexin biosynthesis to repress anti-fungal defense (S9 Data) [32]. However, no strong correlation has been found between trex516 and *CYP71A13* in our dataset, showing that this regulatory pair does not show photoperiodic patterns in our conditions (S30 Fig).

#### Correlation between transposable elements and tRFs.

In plants, tRFs are known to regulate the expression of transposable elements (TEs) [30,57]. To identify potential tRF-TE regulatory pairs, we utilized the mRNA-seq dataset to search for young and intact TE copies in the Arabidopsis genome using RepeatMasker (TAIR10-annotated TE loci > 50% length to original TE and < 10% divergence; S10 Data; **Materials and Methods**). Next, we quantified the expression levels of TEs at both a copy level and at a family level (S11 Data). In total, we detected the expression of 134 TE copies, distributed across 103 TE families; of these, 65 TE copies and 48 TE families were differentially expressed across photoperiods (DESeq2 LRT; adjusted-*p*-value < 0.01; S5 Data and S31 Fig). We clustered differentially expressed TE copies and families into 6 (TEC1–6 for TE copies) and 4 groups (TEF1–4 for TE families), respectively (S32 Fig). We note that a ShD-induction at ZT12 can be seen in the TEC3 group of TE copies (S32 Fig). However, we did not obverse a grouping of expression pattern at a superfamily level, as major superfamilies including Copia and Helitron are scattered across clusters.

Next, we used psRNATarget to predict putative candidate TE targets of tRFs [58] (S12 Data). The 65 TE copies and 2480 tRFs that were differentially-expressed were used as input to predict possible tRF-TE bindings. psRNATarget identified 505′ tRF-TE-copy pairs where both RNAs are differentially regulated, consisting of 282 tRFs and 57 TE copies (S13 Data). Of these 505 pairs, 273 pairs show statistical significant correlations in at least one photoperiodic condition or all three (adjusted-*p*-values < 0.05), consisting of 150 tRFs and 51 TEs (S13 Data). To ask if the tRF-TE pairs show stronger positive or negative correlation in a particular photoperiod, we inspected the distribution of correlation coefficients and the *p*-value distribution of correlation tests in a manner similar to the analysis of miRNA-mRNA pairs (S33 and S34 Figs). Q-Q plots show that *p*-values are smaller than the expected distribution, suggesting the expression patterns of the tRF-TE pairs are non-random (S33 Fig). In general, we did not observe a considerable photoperiodic bias towards positive or negative correlation for the tRF-TE pairs (S34 Fig). Of the 273 pairs, the most numerous significantly correlated combinations are tRF-Tyr^*GTA*^-Copia, tRF-Ser^*AGA*^-Gypsy and tRF-Glu^*CTC*^-Copia, at 31, 17 and 15 pairs (S13 Data). This is not unexpected, because Copia and Gypsy are the most abundant TE superfamilies among differentially expressed TEs (S32 Fig). These tRF and TE types may represent valuable targets for future studies of photoperiodic TE regulations.

#### Expression of tRF-related genes.

Several genes encoding for miRNA processing proteins show photoperiodic expression in the co-sequenced mRNA dataset (S35 Fig). For instance, *HUA ENHANCER 1* (*HEN1*) and *HYPONASTIC LEAVES 1* (*HYL1*) encode a methyltransferase and a double-stranded DNA-binding protein, respectively, that promote miRNA stability through 2′-O-methylation [59]. Loss of function mutations in *HEN1* or *HYL1* leads to decrease in total miRNA abundance. In the co-sequenced mRNA-seq, *HEN1* exhibits higher LgD expression at ZT12, and *HYL1* shows slightly higher ShD levels at ZT12. In contrast, *SERRATE* (*SE*), which encodes a zinc-finger-containing protein crucial to processing pri-miRNA into miRNA, and thus the up-regulation of miRNA levels [60], shows a ZT08-specific trough but no photoperiodic change (adjusted-*p* ≈ 0.0122) (S35 Fig). Overall, we did not observe a uniform regulation across miRNA processing genes, which would be suggestive of photoperiodic miRNA biogenesis. Nevertheless, how the photoperiodic expression of individual miRNA processing genes may affect the downstream sRNA levels remain a key question in understanding photoperiodism.

The biogenesis of tRFs involves a family of RNase T2 proteins that include RIBONUCLEASE 1/2/3 (RNS1/2/3) [32,61,62]. RNS1 is required for the generation of short 5′-tRF-Ala/Gly under phosphate starvation conditions in Arabidopsis [61], while RNS2 generates wounding-induced 16 nt 5′-tRF-Ala [62]. In the co-sequenced mRNA dataset, we detected weak LgD-induction for both *RNS1* and *RNS2*, at ZT16 and ZT08, respectively, but no photoperiodic regulation for *RNS3* (S36 Fig). Short 16 nt 5′-tRF-Ala are observable in T7, while 19 nt 5′-tRF-Gly^*TCC*^ are in T11. Both T7/11 exhibit higher levels in LgD: T7 shows LgD-specific ZT16 peak (S17 Fig), while T11 generally shows higher LgD levels at ZT8-ZT20 (S20 Fig), showing that *RNS1/2* and the associated tRF types are both LgD-induced.

### The Photo-Graph database expands to sRNA data visualization and comprehensive mRNA analyses

Photo-Graph is an online database of photoperiodic time course mRNA-sequencing data generated in our previous work [13]. In this study, we expand the Photo-Graph database to encompass the sRNA-sequencing data presented herein. The updated Photo-Graph has a new interface and allows the search for both miRNAs and tRFs by their miRBase and trex identifiers, respectively. The users may also search for miRNA and tRF sequences. For miRNAs, literature reported target mRNAs are also included. Lastly, the mRNA normalizations presented in here are also available, in addition to our previous mRNA calculations.

In summary, the Photo-Graph, as well as this mRNA-sRNA-co-sequencing dataset, aim to facilitate the discovery of new photoperiodic or diurnal gene regulatory mechanisms. The availability of the sequencing data at NCBI-GEO (accession GEO GSE221846 & GSE289155) coupled with the Photo-Graph interface would allow convenient analysis or re-analysis of the mRNA and sRNA data to answer biological questions towards photoperiodism or diurnal gene regulation.

## Discussion

In plants, miRNAs and likely tRFs represent a regulatory layer separate from TFs/mRNAs and play diverse roles in photoperiodic growth and development. Nevertheless, how photoperiod affects the changes in their levels within a daily cycle has not been extensively studied. This time-course study provides a comprehensive characterization of 83 and 2480 photoperiodically regulated miRNAs and tRFs, respectively (Figs 1B and 3A). We further exploited our previously co-sequenced mRNA dataset to search for correlated miRNA-mRNA and tRF-TE pairs, in an effort to understand how sRNAs may contribute to photoperiodic biological processes through a correlative study.

We identified three miRNAs with significant correlated mRNA targets: miR398a, miR775 and miR400. miR398a showed high negative correlation with *CSD1*/*2* and *CCS*. miR398bc also shows similar pattern with miR398a. It is notable that miR398a and the targets showed the highest correlation in the whole dataset. Given that this study used bulk-sRNA-seq on whole seedling tissues and has limited capacity in detecting tissue specific miRNA or tRF regulations, an exceptionally high correlation in miR398 and the targets may suggest two possibilities: miR398 and the target mRNAs may exist at high concentration at a specific tissue, or miR398 regulate targets in most major tissues. The latter possibility aligns with reports which show with GUS reporters that miR398bc generally show high expression in most seedling tissues, even though miR398a exhibits low expression [41,63]. Interestingly, expressions of the miR398-module suggest that a stress response similar to oxidative stress, drought stress, pathogen infection or copper deficiency may be triggered under shorter photoperiods [64]. Future studies may link a short photoperiod response to one of these responses.

miR775 is a known target of HY5 regulation in light responses [43,44]. Herein, we showed that miR775 displays a photoperiodic rhythm, where induction is observed at light-to-dark time points and repression at dark-to-light time points (Fig 2D). Interestingly, of the eight miRNAs suggested to be controlled by HY5 [44], *i.e.* miR156d, miR172b, miR402, miR408, miR775, miR858, miR869 and miR1888 (S37 Fig), only miR775 was observed to show this photoperiodic pattern; this can be caused by a number of possible mechanisms: for instance, HY5 may regulate miR775 separately from other miRNAs under our growth conditions, or that other pathways regulate miR775 to yield the photoperiodic pattern. Although the strong negative correlation in the miR775-*KFB* pair may suggest direct *KFB* regulation by miR775, it is also possible that both miR775 and *KFB* are separately controlled by HY5, and further investigation is required to confirm these hypotheses. Furthermore, the identification of this characteristic pattern of miR775 suggests that HY5 is a regulator of sRNA level across different photoperiod conditions.

The role of miR400 in defense response and control of ROS as a regulator of two pentatricopeptide-repeat-protein-encoding genes, *PENTATRICOPEPTIDE REPEAT 1/2* (*PRR1/2*), has been well-studied [46,47]. Yet, in this study we detected only weak correlation in the miR400-*PRR1/2* pairs, but strong positive correlation in the miR400-*MTSF4* pair, albeit in LgD photoperiod only (Fig 2E). *MTSF4* is a known anterograde target of miR400 under heat stress response, where down-regulation of miR400 causes upregulation of *MTSF4*. [48]. On one hand, the observation of correlation only in LgD suggests a potential preemptive thermotolerance mechanism triggered by LgD photoperiod, which is indicative of heat; on the other hand, the positive correlation contradicts with the inverse regulatory relationship mentioned in Jung et al (2023) [48]. Nevertheless, we note that an essential gene like *MTSF4* may potentially benefit from a buffering mode of miRNA regulation, instead of a clearance mode, which is more likely to cause a steady-state level of *MTSF4* low enough to trigger growth defects. This may serve as a hypothetical explanation of why positive, instead of negative, correlation was observed.

This study did not identify some miRNA-mRNA pairs that are known to control plant growth and photoperiodism, notably those involving miR156 and miR172, which play major roles in vegetative phase transition and flowering, respectively [20,33]. Both miR156 and miR172 are known to display a change in levels across a developmental time scale. Notably, this study was specifically designed to identify sRNAs that show photoperiodic regulations in a diel time scale. Therefore, it may have limited sensitivity in detecting gradients of miRNA levels that may occur over a multi-day time course. Nevertheless, this work identified that miRNA rhythms that may exist in a diel photoperiodic time scale, as in the case of miR398, in addition to known miRNA gradients in a developmental time scale.

This study identified 20 clusters that represent diverse patterns of photoperiodic tRFs. Intriguingly, in the tRF group T17 we observed a high abundance (> 90% of total reads) of small 5′ tRFs assigned to a tRNA of undetermined amino acid isotype on the tREX database (S26 Fig) [36]. Fragments from this tRNA are also seen in T7 (S16 Fig), and to a lesser extent in T3 (S12 Fig). The genomic locus that produces this tRNA is annotated as AT1G57710 on TAIR10, but the associated tRFs have not been reported to our knowledge [65]. These tRFs are all derived from the 5′ end of tRNA-AT1G57710, are mostly 15–20 nt long. Given the relatively high abundance of these tRFs in this dataset, they may be of considerable interest in future studies of sRNA-mediated photoperiodism.

To explore a potential biological function of tRFs, we quantified TE expression in the mRNA-seq dataset, and identified correlated candidate tRF-TE pairs. *ATHILA6A*-family TEs have been reported to be a target of 19 nt tRF-Met^*CAT*^ in Arabidopsis inflorescence [30]. In this study, the only tRF-Met^*CAT*^-*ATHILA6A* pair we observed consisted of the 21-nt long trex8859 (S38 Fig), showing that the reported regulation was not detectable in the context of this study. Although some TEs have been reported to be responsive to abiotic signals, such as *ONSEN* (*ATCOPIA78*) and *ATCOPIA35* [66], we did not detect photoperiodic expression of these reported TEs. We also note that in this study we focused on relatively high-confidence TEs with high expressions by using RepeatMasker to assess the age and intactness of individual TE copies (Materials and Methods). A limitation of this approach is that shorter TEs, or TEs of specific superfamilies more difficult to annotate were not covered in this study (S31 Fig and S10 Data). For example, the Helitron superfamily TEs do not generate terminal inverted repeats or target site duplications, and specific annotators have been developed for them [67,68]. In this study, we note that the majority of TAIR-annotated Helitron TEs were not quantified due to alignment limitations in RepeatMasker (S31 Fig and S10 Data). Nevertheless, the identification of photoperiodic TE patterns, and their correlation with potential tRF regulators in this study, may serve as the basis of future investigations on the role of tRF in TE regulation. Superfamily-specific annotation pipelines may be used to further characterize the photoperiodic expression of TE copies.

This co-sequencing dataset also allowed us to inspect the expression patterns of genes encoding proteins involved in tRF biogenesis, including *RNS1*/*2*/*3*. Notably, we identified that both *RNS1*/*2* show higher levels in LgD, while 16 nt 5′-tRF-Ala and 19 nt 5′-tRF-Gly^*TCC*^ that are generated by RNS1/2 also show LgD-induction [61,62]. This coincidence in expression patterns between RNS1 and the associated tRFs may indicate a potential LgD-specific tRF biogenesis mechanism. Furthermore, we note that *RNS1* expression is inducible by phosphate starvation [69]. Our detection of a phosphate-starvation-induced tRF in T11 (trex1082) may support the speculation that a phosphate starvation signal is present at the late long day photoperiod.

In both miRNAs and tRFs, we identified expression patterns that strongly resemble the characteristic ShD-induced and LgD-induced MDLM genes [9,10]. This includes miRNA and tRF clusters M1/T1/T2/T4/T6 for ShD-MDLM patterns, and T12/T14 for LgD-MDLM patterns. The pattern of miRNA cluster M6 also slightly resembles LgD-MDLM, showing high levels in the mid-day of LgD and EqD. Overall, the strong resemblance of patterns suggest that MDLM, or MDLM-controlled genes are upstream of these clusters. On the other hand, there are miRNA and tRF patterns that are unlikely to be regulated by MDLM. For instance, the photoperiodic pre-dusk-peaking pattern of miR775 is likely controlled by HY5, even though further investigation is required for confirmation. Other possible known photoperiodic mechanisms are also possible drivers of the observed patterns. *e.g.,* the ShD-specific ZT24 dip in T3 can be partially explained by the accumulation of a regulator in the long nighttime of a ShD photoperiod, which would align with the nighttime accumulation of phytochrome A proteins [70].

The identification of numerous photoperiodic patterns in both miRNAs and tRFs suggests the presence of multiple sRNA regulatory mechanisms, and further investigation into these individual mechanisms may greatly expand our understandings into how plants attune to light signals and seasonal events. In a wider context, further understanding of plant photoperiodism provide a new perspective in crop improvement and mitigation of climate change.

## Materials and methods

### Plant materials for sequencing

The plant materials and RNA extraction process were identical to those described in [13] as they originated from the same samples. Briefly, Arabidopsis Col-0 seeds were sterilized before being sown onto 1/2 Murashige and Skoog medium plates (2.15 g/L Murashige and Skoog medium at pH 5.6, Cassion Laboratories, cat. #MSP01, and 0.8% bacteriological agar, AmericanBio cat. #AB01185) lined with sterile filter papers. For germination, seeds were stratified in dark at 4° C for 48 hours before being transferred to a growth chamber under 12L:12D photoperiod at 22° C and light intensity of 130 μ. After germination, seedlings were kept in the same condition for 10 days. On day 11, the seedlings were transferred to 16L:8D, 12L:12D, or 8L:16D photoperiod. On day 13, whole seedlings including shoots and roots were harvested and snap-frozen in liquid nitrogen.

### Library preparation of small RNA sequencing

Total RNA was extracted from seedling shoot and roots using TRIzol reagent (ThermoFisher, 15596026) according to manufacturer’s protocol. Residual DNA was removed with RNase-free DNase (QIAGEN, 79254). A treatment with phenol:chloroform:isoamyl-alchohol mixture (25:24:1; ThermoFisher, AM9730) followed by precipitation with 3 M sodium acetate solution was used to remove protein contaminants. The resulted total RNA samples were sent to Yale Center for Genome Anlaysis for library preparation. The quality of total RNA samples was checked with Agilent Bioanalyzer for an RNA integration number ≥ 7.0. RNA size selection was performed with a 15% denaturing polyacrilamide gel and bands representing RNAs of 15–40 base pairs were excised. NEBNext Low-bias Small RNA Library Prep Kit (New England Biolabs cat number E3420L) was used for library construction for accurate representation of both miRNAs and tRFs; libraries were constructured following the manufacturer’s protocol. The libraries were sequenced on the Illumina NovaSeq6000 platform (S1 flow cells) to generate single-end reads.

### Processing of bulk small RNA sequencing data

Read quality was checked with FastQC (v0.11.9) [71]. Sequencing adapters and low quality reads were removed from the raw read file using cutadapt (v3.4) with the following parameters: -q 10,10 [72]. tRF and miRNA alignments were performed separately. First, highly stringent mapping was performed for tRFs due to the tiling nature of the fragments and the high sequence similarity between tRNAs of origin. Known Arabidopsis tRFs (1,544,607 in total) were downloaded from the tREX database (2021 release) [36]. Fragments in the tREX database that cannot be distinguished from each other due to an ambiguous base N were excluded. No tREX database sequence is identical to a known miRNA. Bowtie2 (v2.5.1) was used to align reads with the following parameters: -a --end-to-end --score-min L,0,-0.6 --norc --np 0 --rdg 9999,9999 --rfg 9999,9999 [73]. SAMTools and awk were used to retrieve alignments where the reads covered the entirety of the sRNA annotations without mismatch (*i.e.,* NM = 0) and without gap (*i.e.,* the read length is identical to the annotation length) [74]. Any unmapped reads were used for the subsequent miRNA mapping, which was less stringent. For miRNA alignment, sequences of known mature Arabidopsis miRNAs (428 in total, but only 350 unique sequences) were downloaded from miRBase (release 22.1) [35]. Bowtie2 (v2.5.1) was used to align reads with the following parameters: -k 10 --end-to-end --norc -N 1 -L 15 [73]. SAMTools were used to retrieve the best alignment for each read with the mapping quality filter -q 20 [74]. Finally, all unaligned reads were subsequently mapped to the TAIR10 (release 57) annotation from Ensembl with Bowtie2 using default parameters [75].

### Processing of bulk messenger RNA sequencing (mRNA-seq) data

mRNA-seq data was obtained from record GSE221846 in NCBI GEO database [13]. Raw reads were processed with trimmomatic (v0.39) with the following parameters: ILLUMINACLIP:file.fa:2:30:10:1:TRUE SLIDINGWINDOW:4:20 LEADING:5 TRAILING:5 MINLEN:36 [76]. Quality check was performed with FastQC (v0.11.9) [71]. Transcripts were quantified with Salmon (v1.4.0) with the following parameters: -l A --numGibbsSamples 50 [77]. The Salmon index was built from genomic sequences with decoys from cDNA, using the TAIR10 (release 57) annotation from Ensembl [75].

### Quantification of Transposable Element (TE) Expression in mRNA-seq

Annotation of TE copies were downloaded from TAIR (TAIR10 March 8th, 2024 version) [65]. The RepbaseUpdate Arabidopsis TE library from The Unit Resource Genomics Info (URGI) database was downloaded from the official website (https://urgi.versailles.inrae.fr/Data/Transposable-elements/Arabidopsis; accessed January 2026) [78]. RepeatMasker function calcDivergenceFromAlign.pl was used to calculate the divergence of individual TE copies in the TAIR genome [79]. Relatively young and intact TE copies are defined as copies that satisfy all of the three requirements are used for downstream analyses: 1) matched to a TE in the RepbaseUpdate TE library by RepeatMasker; 2) at least 50% of the length of the original TE; 3) under 10% divergence as calculated by RepeatMasker. 4374 of the 31189 TE copies in the TAIR10 annotation were considered young and intact by these criteria.

TE expression was quantified at two levels: a family level and a copy level. For both levels, STAR was used to align the mRNA-seq reads to the Arabidopsis genome with the following parameters: --outFilterMultimapNmax 100 --winAnchorMultimapNmax 100 [80]. For family-level-quantification, TEtranscript [81] was used to quantify expression with the parameters --format BAM --mode multi --stranded reverse; notably, only young and intact TE copies were quantified. For copy-level-quantification, the TElocal functionality from the TEtranscript suite was used with the following parameters: --mode uniq --stranded reverse. The unique-mapping mode was used for copy-level quantification to ensure accuracy. The order and superfamily classifications of TEs follow the TAIR annotation. The ambiguous “TSCL-LINE?” classification was treated as “Unassigned” in this study.

### Differential expression analysis of mRNA-seq and sRNA-seq data

All R packages mentioned hereafter, unless otherwise specified, were run with R (v4.4.0) [82]. For mRNA-seq, DESeq2 (v1.42.1) was used for data normalization and differential expression analysis [83]. All steps were performed with default parameters unless otherwise specified. Salmon-quantified transcripts were merged into genes with tximport (v1.26.1) [84]. Only genes with at least 10 units in at least 3 samples were retained. For transposable elements, an additional filter of at least 100 FPKM in at least 3 samples were used to remove long TEs with low read counts. The LRT method of DESeq2 was used to identify photoperiodic genes: likelihood ratio=−2ln(L(~Time+Photoperiod+Time:Photoperiod)/L( Time+Photoperiod)). Differentially expressed genes are defined with adjusted-*p* < 0.01 after independent hypothesis weighing [85]. For sRNA-seq, the procedures were identical with the exception that Bowtie2-generated read counts were directly imported into DESeq2 without tximport. miRNAs and tRFs were processed separately. Read counts of mRNAs and sRNAs were output in two formats for downstream analyses: read per million (RPM) and regularized log transformed (RLD) values. RPM provides a readily understandable metric to quantify the abundance of reads across libraries, while the RLD procedure yields a variance-stabilizing effect that is favorable for clustering analyses and the observation of outlier data points [83]. The use of either value is clearly stated in each figure.

### Clustering and heat map visualization

Clustering and heat map visualizations were generated by the ComplexHeatmap software (v.2.20.0) [86]. For all RNAs, the Manhattan distance metric and the complete hierarchical clustering method were used. The clustering was performed over scaled RNA levels across 54 samples and the row_split argument was used to yield the final number of clusters. Scaling was performed gene-wise with Z-score normalization across all conditions, except for figure S9 Fig where Z-score normalization was performed separately for each photoperiod. For each RNA, the daily expression integral (DEI) for each photoperiod, *i.e.,* *DEI_LgD_*, *DEI_EqD_* or *DEI_ShD_*, refers to the sum of expression value in RLD in the respective photoperiod. The relative DEI ratio (rDEI ratio) for each RNA was visualized as a tricolored bar plot in heat maps for easy inspection of the expression level in each photoperiod. The rDEI ratio for each gene was calculated by 1) separately raising the three separate DEIs to the power of 12 for easy visualization, and 2) dividing each of the photoperiodic sums by the sum of all three sums. For example, the height of the red bar representing DEILgD is given by *i.e.,* $\frac{DEI_{LgD}^{12}}{DEI_{LgD}^{12}+DEI_{EqD}^{12}+DEI_{ShD}^{12}}$.

### Testing correlations of miRNA-mRNA pairs

Known miRNA-mRNA pairs were downloaded from three databases: PmiREN2.0 [39], miRTarBase 2022 [37] and TarDB (accessed July 2024) [38]. For every pair where both RNAs are differentially regulated by photoperiod (adjusted-*p*-value < 0.01), a two-sided Pearson’s correlation test was performed. The correlations were calculated on a per-sample basis, as individual biological samples were paired between the mRNA-sequencing and sRNA-sequencing. Correlations were tested four times, one across 54 samples, and three across 18 samples of each photoperiod. *p*-values were adjusted with the Benjamini-Hochberg procedure separately for each of the four tests [87]. A threshold of adjusted-*p*-value < 0.05 was used. The tests were performed on RLD values. The correlation between miR398a-3p and *CSD2* (*COPPER/ZINC SUPEROXIDE DISMUTASE 2*; *AT2G28190*) was separately tested, and the *p*-value was not adjusted. Faceted line and dot plots of RNA profiles were generated by ggplot2 (v3.5.1) [88] and ggpubr (v0.6.0) [89]. RLD values were used for plotting. For quantile-quantile (Q-Q) plots, the unadjusted-*p*-values were used. The R function ppoints() was used to generate the expected *p*-value distribution.

### Analyses of tRNA fragment size and origin tRNAs

The size of each tRNA fragment was calculated from the tREX FASTA file using Biostrings [90]. The FASTA sequences of tRNA and the flanking regions were downloaded from tREX database [36]. rBLAST was used to map fragments onto the tRNA sequences with flanking regions [91]. Arguments were -task blastn-short -strand plus. All matches with the lowest *e*-value stratum were considered as matches. tRNA fragments that match to multiple tRNAs of different isotypes or different anticodons were categorized as a separate tRF class. To calculate the proportion of each tRF class in a tRF cluster, RPM (read per million) values of all tRFs in each tRFs class were summed. A stacked bar chart of circular coordinates was plot from the sums using ggplot2 (v3.5.1) [88]. To generate bar charts that display the mapping of tRFs on tRNAs or the flanking regions, the BLAST mapping positions were scaled by the RPM sum of each tRF class. The mapping positions were binned into 100 percentiles (0–99).

### Prediction of tRF targets

psRNATarget (version 2, 2017) was used to predict transposable element (TE) targets of tRFs using default parameters (https://www.zhaolab.org/psRNATarget; accessed January 2026) [92]. For tRF-TE correlation at a TE copy level, all differentially regulated (adjusted-*p*-value < 0.01) tRFs and young TE copies were used: this included 65 TE copies and 2480 tRFs. All pairs with expectation score lesser than or equal to 5.0 were used: this generated 505 pairs. All analyses of correlations, including generation of quantile-quantile plots and calculation of *p*-values, for tRF-TE pairs follow those of miRNA-mRNA pairs. For tRF-TE correlations at a family level, all differentially regulated (adjusted-*p*-value < 0.01) TE families (48 TE families) with at least one copy to that is targeted by the tRF were analyzed (38 out of 48 TE families).

## Supporting information

S1 Fig Pie charts illustrating the number of detected non-transposable element photoperiodic mRNAs. (EPS)

S2 Fig Heat map of the clustering of photoperiodic non-transposable element mRNAs. Main body: heat map shows the clustering of 16,030 photoperiodic non-transposable element mRNAs, into twenty four groups (C1 - C24; likelihood ratio test: adjusted-*p* < 0.01); Photoperiodic DEI Proportion: stacked bar chart of the proportional daily expression integral (DEI) of each gene, raised to the power of twelve for visualization (red = LgD; orange = EqD; blue = ShD); total expression: heat map of the total expression in RLD-normalized read counts.
(EPS)

S3 Fig Distribution of Pearson’s correlation coefficients of miRNA-mRNA pairs. Histograms show the distributions in all samples (upper left), LgD-only samples (lower left), EqD-only samples (upper right) and ShD-only samples (lower right). Pearson’s correlation range from 1, indicating complete positive correlation, to -1, indicating complete inverse correlation. A coefficient of 0 indicates no correlation.
(EPS)

S4 Fig Quantile-quantile plot of the *p*-values for Pearson’s correlation test for miRNA-mRNA pairs. The observed unadjusted-*p*-value are plotted against the expected *p*-value for the Pearson’s correlation test for each miRNA-mRNA pair in an analysis with (A) LgD samples only, (B) EqD samples only, (C) ShD samples only and (D) across all samples in LgD, EqD and ShD. Axis are in -log_10_ scale. Straight line indicates the values where observed *p*-values equal to expected *p*-values. Red and blue dots indicate positively and negatively correlated pairs, respectively.
(EPS)

S5 Fig Violin plot of the Pearson’s correlation coefficient for time-shifted miRNA-mRNA pairs. Distributions of Pearson’s correlation coefficients (PCCs) of tRF-TE pairs calculated using LgD samples only, EqD samples only, ShD samples only, and all samples are represented by the gray, red, orange and blue violins. Individual dots indicate individual pairs.
(EPS)

S6 Fig Quantile-quantile plot of the *p*-values for Pearson’s correlation test for time-shifted miRNA-mRNA pairs. The observed unadjusted-*p*-value are plotted against the expected *p*-value for the Pearson’s correlation test for each miRNA-mRNA pair in an analysis with (A) LgD samples only, (B) EqD samples only, (C) ShD samples only and (D) across all samples in LgD, EqD and ShD. Axis are in -log_10_ scale. Straight line indicates the values where observed *p*-values equal to expected *p*-values. Red and blue dots indicate positively and negatively correlated pairs, respectively.
(EPS)

S7 Fig Expression levels of selected time-shifted pairs of miRNAs and mRNAs. Expression levels of selected miRNAs and mRNAs. Lines indicate mean and colors indicate photoperiod (red = LgD; orange = EqD; blue = ShD). Data points are represented in squares, triangles or circles; the shapes represent the pairing of total RNA samples between s/mRNA-co-sequencing. Asterisk(s) (*) indicates statistical significance of the test of Pearson’s correlation coefficient (PCC). Single (*) and double (**) asterisks indicate adjusted-*p* < 0.05 and ** adjusted-*p* < 0.01, respectively. Expressions are in regularized-log-transformed values (RLD).
(EPS)

S8 Fig Expression levels of selected pairs of miRNAs and mRNAs. Expression levels of selected miRNAs and mRNAs. Lines indicate mean and colors indicate photoperiod (red = LgD; orange = EqD; blue = ShD). Data points are represented in squares, triangles or circles; the shapes represent the pairing of total RNA samples between s/mRNA-co-sequencing. Asterisk(s) (*) indicates statistical significance of the test of Pearson’s correlation coefficient (PCC). Single (*) and double (**) asterisks indicate adjusted-*p* < 0.05 and ** adjusted-*p* < 0.01, respectively. Expressions are in regularized-log-transformed values (RLD).
(EPS)

S9 Fig Heat map of the clustering of photoperiodic tRFs with independent scaling for each photoperiod. Main body: heat map shows the clustering of 2,480 photoperiodic tRFs into twenty four groups (C1 - C24; likelihood ratio test: adjusted-*p* < 0.01). The clustering is identical to Fig 3B, but the heat map coloring indicates the Z-score-normalized expression values that are scaled independently for each photoperiod for an alternative visualization. Photoperiodic DEI Proportion: stacked bar chart of the proportional daily expression integral (DEI) of each gene, raised to the power of twelve for visualization (red = LgD; orange = EqD; blue = ShD); total expression: heat map of the total expression in RLD-normalized read counts.
(EPS)

S10 Fig Summary of tRF properties for cluster T1. **A)** Mean abundance level of tRFs in the cluster. For every tRF, the relative level is calculated by Z-score normalization. Error bars indicate standard error of the mean. The colors indicate photoperiod (red = LgD; orange = EqD; blue = ShD). tRF number: the number of unique tRF sequences in the cluster. **B)** Pie chart showing the proportion of each tRF class in the cluster by read count (in reads per million, *i.e.,* RPM). Only the top 3 abundant tRF classes by RPM are labeled. **C)** Bar charts showing the total RPM level of each tRF class by fragment size (in number of nucleotides, *i.e.,* nt). Vertical black line separates the shorter tRFs (15–29 nt) on the left from the longer tRFs (30–48 nt) on the right. **D)** Bar charts showing the mapping of tRFs onto the tRNA gene locus for each tRF class. The left, center and right X-axes show the percentile position of the 5′ 100 flaking region, mature tRNA and 3’ flanking region. Mature tRNAs vary in length and therefore the length of each mature tRNA was split into 100 percentiles. The y-axis value indicates the number of tRFs that map to the percentile by BLAST in RPM. The graphs in (B), (C) and (D) are color-coded by the amino acid of the origin tRNA, following the scheme in Fig 3 and Fig 4. Unlabeled tRF classes are in light grey. tRF classes that involve multiple tRNA isotypes are in dark gray.
(EPS)

S11 Fig Summary of tRF properties for cluster T2. **A)** Mean abundance level of tRFs in the cluster. For every tRF, the relative level is calculated by Z-score normalization. Error bars indicate standard error of the mean. The colors indicate photoperiod (red = LgD; orange = EqD; blue = ShD). tRF number: the number of unique tRF sequences in the cluster. **B)** Pie chart showing the proportion of each tRF class in the cluster by read count (in reads per million, *i.e.,* RPM). Only the top 3 abundant tRF classes by RPM are labeled. **C)** Bar charts showing the total RPM level of each tRF class by fragment size (in number of nucleotides, *i.e.,* nt). Vertical black line separates the shorter tRFs (15–29 nt) on the left from the longer tRFs (30–48 nt) on the right. **D)** Bar charts showing the mapping of tRFs onto the tRNA gene locus for each tRF class. The left, center and right X-axes show the percentile position of the 5′ 100 flaking region, mature tRNA and 3’ flanking region. Mature tRNAs vary in length and therefore the length of each mature tRNA was split into 100 percentiles. The y-axis value indicates the number of tRFs that map to the percentile by BLAST in RPM. The graphs in (B), (C) and (D) are color-coded by the amino acid of the origin tRNA, following the scheme in Fig 3 and Fig 4. Unlabeled tRF classes are in light grey. tRF classes that involve multiple tRNA isotypes are in dark gray.
(EPS)

S12 Fig Summary of tRF properties for cluster T3. **A)** Mean abundance level of tRFs in the cluster. For every tRF, the relative level is calculated by Z-score normalization. Error bars indicate standard error of the mean. The colors indicate photoperiod (red = LgD; orange = EqD; blue = ShD). tRF number: the number of unique tRF sequences in the cluster. **B)** Pie chart showing the proportion of each tRF class in the cluster by read count (in reads per million, *i.e.,* RPM). Only the top 3 abundant tRF classes by RPM are labeled. **C)** Bar charts showing the total RPM level of each tRF class by fragment size (in number of nucleotides, *i.e.,* nt). Vertical black line separates the shorter tRFs (15–29 nt) on the left from the longer tRFs (30–48 nt) on the right. **D)** Bar charts showing the mapping of tRFs onto the tRNA gene locus for each tRF class. The left, center and right X-axes show the percentile position of the 5′ 100 flaking region, mature tRNA and 3’ flanking region. Mature tRNAs vary in length and therefore the length of each mature tRNA was split into 100 percentiles. The y-axis value indicates the number of tRFs that map to the percentile by BLAST in RPM. The graphs in (B), (C) and (D) are color-coded by the amino acid of the origin tRNA, following the scheme in Fig 3 and Fig 4. Unlabeled tRF classes are in light grey. tRF classes that involve multiple tRNA isotypes are in dark gray.
(EPS)

S13 Fig Summary of tRF properties for cluster T4. **A)** Mean abundance level of tRFs in the cluster. For every tRF, the relative level is calculated by Z-score normalization. Error bars indicate standard error of the mean. The colors indicate photoperiod (red = LgD; orange = EqD; blue = ShD). tRF number: the number of unique tRF sequences in the cluster. **B)** Pie chart showing the proportion of each tRF class in the cluster by read count (in reads per million, *i.e.,* RPM). Only the top 3 abundant tRF classes by RPM are labeled. **C)** Bar charts showing the total RPM level of each tRF class by fragment size (in number of nucleotides, *i.e.,* nt). Vertical black line separates the shorter tRFs (15–29 nt) on the left from the longer tRFs (30–48 nt) on the right. **D)** Bar charts showing the mapping of tRFs onto the tRNA gene locus for each tRF class. The left, center and right X-axes show the percentile position of the 5′ 100 flaking region, mature tRNA and 3’ flanking region. Mature tRNAs vary in length and therefore the length of each mature tRNA was split into 100 percentiles. The y-axis value indicates the number of tRFs that map to the percentile by BLAST in RPM. The graphs in (B), (C) and (D) are color-coded by the amino acid of the origin tRNA, following the scheme in Fig 3 and Fig 4. Unlabeled tRF classes are in light grey. tRF classes that involve multiple tRNA isotypes are in dark gray.
(EPS)

S14 Fig Summary of tRF properties for cluster T5. **A)** Mean abundance level of tRFs in the cluster. For every tRF, the relative level is calculated by Z-score normalization. Error bars indicate standard error of the mean. The colors indicate photoperiod (red = LgD; orange = EqD; blue = ShD). tRF number: the number of unique tRF sequences in the cluster. **B)** Pie chart showing the proportion of each tRF class in the cluster by read count (in reads per million, *i.e.,* RPM). Only the top 3 abundant tRF classes by RPM are labeled. **C)** Bar charts showing the total RPM level of each tRF class by fragment size (in number of nucleotides, *i.e.,* nt). Vertical black line separates the shorter tRFs (15–29 nt) on the left from the longer tRFs (30–48 nt) on the right. **D)** Bar charts showing the mapping of tRFs onto the tRNA gene locus for each tRF class. The left, center and right X-axes show the percentile position of the 5′ 100 flaking region, mature tRNA and 3’ flanking region. Mature tRNAs vary in length and therefore the length of each mature tRNA was split into 100 percentiles. The y-axis value indicates the number of tRFs that map to the percentile by BLAST in RPM. The graphs in (B), (C) and (D) are color-coded by the amino acid of the origin tRNA, following the scheme in Fig 3 and Fig 4. Unlabeled tRF classes are in light grey. tRF classes that involve multiple tRNA isotypes are in dark gray.
(EPS)

S15 Fig Summary of tRF properties for cluster T6. **A)** Mean abundance level of tRFs in the cluster. For every tRF, the relative level is calculated by Z-score normalization. Error bars indicate standard error of the mean. The colors indicate photoperiod (red = LgD; orange = EqD; blue = ShD). tRF number: the number of unique tRF sequences in the cluster. **B)** Pie chart showing the proportion of each tRF class in the cluster by read count (in reads per million, *i.e.,* RPM). Only the top 3 abundant tRF classes by RPM are labeled. **C)** Bar charts showing the total RPM level of each tRF class by fragment size (in number of nucleotides, *i.e.,* nt). Vertical black line separates the shorter tRFs (15–29 nt) on the left from the longer tRFs (30–48 nt) on the right. **D)** Bar charts showing the mapping of tRFs onto the tRNA gene locus for each tRF class. The left, center and right X-axes show the percentile position of the 5′ 100 flaking region, mature tRNA and 3’ flanking region. Mature tRNAs vary in length and therefore the length of each mature tRNA was split into 100 percentiles. The y-axis value indicates the number of tRFs that map to the percentile by BLAST in RPM. The graphs in (B), (C) and (D) are color-coded by the amino acid of the origin tRNA, following the scheme in Fig 3 and Fig 4. Unlabeled tRF classes are in light grey. tRF classes that involve multiple tRNA isotypes are in dark gray.
(EPS)

S16 Fig Summary of tRF properties for cluster T7. **A)** Mean abundance level of tRFs in the cluster. For every tRF, the relative level is calculated by Z-score normalization. Error bars indicate standard error of the mean. The colors indicate photoperiod (red = LgD; orange = EqD; blue = ShD). tRF number: the number of unique tRF sequences in the cluster. **B)** Pie chart showing the proportion of each tRF class in the cluster by read count (in reads per million, *i.e.,* RPM). Only the top 3 abundant tRF classes by RPM are labeled. **C)** Bar charts showing the total RPM level of each tRF class by fragment size (in number of nucleotides, *i.e.,* nt). Vertical black line separates the shorter tRFs (15–29 nt) on the left from the longer tRFs (30–48 nt) on the right. **D)** Bar charts showing the mapping of tRFs onto the tRNA gene locus for each tRF class. The left, center and right X-axes show the percentile position of the 5′ 100 flaking region, mature tRNA and 3’ flanking region. Mature tRNAs vary in length and therefore the length of each mature tRNA was split into 100 percentiles. The y-axis value indicates the number of tRFs that map to the percentile by BLAST in RPM. The graphs in (B), (C) and (D) are color-coded by the amino acid of the origin tRNA, following the scheme in Fig 3 and Fig 4. Unlabeled tRF classes are in light grey. tRF classes that involve multiple tRNA isotypes are in dark gray.
(EPS)

S17 Fig Summary of tRF properties for cluster T8. **A)** Mean abundance level of tRFs in the cluster. For every tRF, the relative level is calculated by Z-score normalization. Error bars indicate standard error of the mean. The colors indicate photoperiod (red = LgD; orange = EqD; blue = ShD). tRF number: the number of unique tRF sequences in the cluster. **B)** Pie chart showing the proportion of each tRF class in the cluster by read count (in reads per million, *i.e.,* RPM). Only the top 3 abundant tRF classes by RPM are labeled. **C)** Bar charts showing the total RPM level of each tRF class by fragment size (in number of nucleotides, *i.e.,* nt). Vertical black line separates the shorter tRFs (15–29 nt) on the left from the longer tRFs (30–48 nt) on the right. **D)** Bar charts showing the mapping of tRFs onto the tRNA gene locus for each tRF class. The left, center and right X-axes show the percentile position of the 5′ 100 flaking region, mature tRNA and 3’ flanking region. Mature tRNAs vary in length and therefore the length of each mature tRNA was split into 100 percentiles. The y-axis value indicates the number of tRFs that map to the percentile by BLAST in RPM. The graphs in (B), (C) and (D) are color-coded by the amino acid of the origin tRNA, following the scheme in Fig 3 and Fig 4. Unlabeled tRF classes are in light grey. tRF classes that involve multiple tRNA isotypes are in dark gray.
(EPS)

S18 Fig Summary of tRF properties for cluster T9. **A)** Mean abundance level of tRFs in the cluster. For every tRF, the relative level is calculated by Z-score normalization. Error bars indicate standard error of the mean. The colors indicate photoperiod (red = LgD; orange = EqD; blue = ShD). tRF number: the number of unique tRF sequences in the cluster. **B)** Pie chart showing the proportion of each tRF class in the cluster by read count (in reads per million, *i.e.,* RPM). Only the top 3 abundant tRF classes by RPM are labeled. **C)** Bar charts showing the total RPM level of each tRF class by fragment size (in number of nucleotides, *i.e.,* nt). Vertical black line separates the shorter tRFs (15–29 nt) on the left from the longer tRFs (30–48 nt) on the right. **D)** Bar charts showing the mapping of tRFs onto the tRNA gene locus for each tRF class. The left, center and right X-axes show the percentile position of the 5′ 100 flaking region, mature tRNA and 3’ flanking region. Mature tRNAs vary in length and therefore the length of each mature tRNA was split into 100 percentiles. The y-axis value indicates the number of tRFs that map to the percentile by BLAST in RPM. The graphs in (B), (C) and (D) are color-coded by the amino acid of the origin tRNA, following the scheme in Fig 3 and Fig 4. Unlabeled tRF classes are in light grey. tRF classes that involve multiple tRNA isotypes are in dark gray.
(EPS)

S19 Fig Summary of tRF properties for cluster T10. **A)** Mean abundance level of tRFs in the cluster. For every tRF, the relative level is calculated by Z-score normalization. Error bars indicate standard error of the mean. The colors indicate photoperiod (red = LgD; orange = EqD; blue = ShD). tRF number: the number of unique tRF sequences in the cluster. **B)** Pie chart showing the proportion of each tRF class in the cluster by read count (in reads per million, *i.e.,* RPM). Only the top 3 abundant tRF classes by RPM are labeled. **C)** Bar charts showing the total RPM level of each tRF class by fragment size (in number of nucleotides, *i.e.,* nt). Vertical black line separates the shorter tRFs (15–29 nt) on the left from the longer tRFs (30–48 nt) on the right. **D)** Bar charts showing the mapping of tRFs onto the tRNA gene locus for each tRF class. The left, center and right X-axes show the percentile position of the 5′ 100 flaking region, mature tRNA and 3’ flanking region. Mature tRNAs vary in length and therefore the length of each mature tRNA was split into 100 percentiles. The y-axis value indicates the number of tRFs that map to the percentile by BLAST in RPM. The graphs in (B), (C) and (D) are color-coded by the amino acid of the origin tRNA, following the scheme in Fig 3 and Fig 4. Unlabeled tRF classes are in light grey. tRF classes that involve multiple tRNA isotypes are in dark gray.
(EPS)

S20 Fig Summary of tRF properties for cluster T11. **A)** Mean abundance level of tRFs in the cluster. For every tRF, the relative level is calculated by Z-score normalization. Error bars indicate standard error of the mean. The colors indicate photoperiod (red = LgD; orange = EqD; blue = ShD). tRF number: the number of unique tRF sequences in the cluster. **B)** Pie chart showing the proportion of each tRF class in the cluster by read count (in reads per million, *i.e.,* RPM). Only the top 3 abundant tRF classes by RPM are labeled. **C)** Bar charts showing the total RPM level of each tRF class by fragment size (in number of nucleotides, *i.e.,* nt). Vertical black line separates the shorter tRFs (15–29 nt) on the left from the longer tRFs (30–48 nt) on the right. **D)** Bar charts showing the mapping of tRFs onto the tRNA gene locus for each tRF class. The left, center and right X-axes show the percentile position of the 5′ 100 flaking region, mature tRNA and 3’ flanking region. Mature tRNAs vary in length and therefore the length of each mature tRNA was split into 100 percentiles. The y-axis value indicates the number of tRFs that map to the percentile by BLAST in RPM. The graphs in (B), (C) and (D) are color-coded by the amino acid of the origin tRNA, following the scheme in Fig 3 and Fig 4. Unlabeled tRF classes are in light grey. tRF classes that involve multiple tRNA isotypes are in dark gray.
(EPS)

S21 Fig Summary of tRF properties for cluster T12. **A)** Mean abundance level of tRFs in the cluster. For every tRF, the relative level is calculated by Z-score normalization. Error bars indicate standard error of the mean. The colors indicate photoperiod (red = LgD; orange = EqD; blue = ShD). tRF number: the number of unique tRF sequences in the cluster. **B)** Pie chart showing the proportion of each tRF class in the cluster by read count (in reads per million, *i.e.,* RPM). Only the top 3 abundant tRF classes by RPM are labeled. **C)** Bar charts showing the total RPM level of each tRF class by fragment size (in number of nucleotides, *i.e.,* nt). Vertical black line separates the shorter tRFs (15–29 nt) on the left from the longer tRFs (30–48 nt) on the right. **D)** Bar charts showing the mapping of tRFs onto the tRNA gene locus for each tRF class. The left, center and right X-axes show the percentile position of the 5′ 100 flaking region, mature tRNA and 3’ flanking region. Mature tRNAs vary in length and therefore the length of each mature tRNA was split into 100 percentiles. The y-axis value indicates the number of tRFs that map to the percentile by BLAST in RPM. The graphs in (B), (C) and (D) are color-coded by the amino acid of the origin tRNA, following the scheme in Fig 3 and Fig 4. Unlabeled tRF classes are in light grey. tRF classes that involve multiple tRNA isotypes are in dark gray.
(EPS)

S22 Fig Summary of tRF properties for cluster T13. **A)** Mean abundance level of tRFs in the cluster. For every tRF, the relative level is calculated by Z-score normalization. Error bars indicate standard error of the mean. The colors indicate photoperiod (red = LgD; orange = EqD; blue = ShD). tRF number: the number of unique tRF sequences in the cluster. **B)** Pie chart showing the proportion of each tRF class in the cluster by read count (in reads per million, *i.e.,* RPM). Only the top 3 abundant tRF classes by RPM are labeled. **C)** Bar charts showing the total RPM level of each tRF class by fragment size (in number of nucleotides, *i.e.,* nt). Vertical black line separates the shorter tRFs (15–29 nt) on the left from the longer tRFs (30–48 nt) on the right. **D)** Bar charts showing the mapping of tRFs onto the tRNA gene locus for each tRF class. The left, center and right X-axes show the percentile position of the 5′ 100 flaking region, mature tRNA and 3’ flanking region. Mature tRNAs vary in length and therefore the length of each mature tRNA was split into 100 percentiles. The y-axis value indicates the number of tRFs that map to the percentile by BLAST in RPM. The graphs in (B), (C) and (D) are color-coded by the amino acid of the origin tRNA, following the scheme in Fig 3 and Fig 4. Unlabeled tRF classes are in light grey. tRF classes that involve multiple tRNA isotypes are in dark gray.
(EPS)

S23 Fig Summary of tRF properties for cluster T14. **A)** Mean abundance level of tRFs in the cluster. For every tRF, the relative level is calculated by Z-score normalization. Error bars indicate standard error of the mean. The colors indicate photoperiod (red = LgD; orange = EqD; blue = ShD). tRF number: the number of unique tRF sequences in the cluster. **B)** Pie chart showing the proportion of each tRF class in the cluster by read count (in reads per million, *i.e.,* RPM). Only the top 3 abundant tRF classes by RPM are labeled. **C)** Bar charts showing the total RPM level of each tRF class by fragment size (in number of nucleotides, *i.e.,* nt). Vertical black line separates the shorter tRFs (15–29 nt) on the left from the longer tRFs (30–48 nt) on the right. **D)** Bar charts showing the mapping of tRFs onto the tRNA gene locus for each tRF class. The left, center and right X-axes show the percentile position of the 5′ 100 flaking region, mature tRNA and 3’ flanking region. Mature tRNAs vary in length and therefore the length of each mature tRNA was split into 100 percentiles. The y-axis value indicates the number of tRFs that map to the percentile by BLAST in RPM. The graphs in (B), (C) and (D) are color-coded by the amino acid of the origin tRNA, following the scheme in Fig 3 and Fig 4. Unlabeled tRF classes are in light grey. tRF classes that involve multiple tRNA isotypes are in dark gray.
(EPS)

S24 Fig Summary of tRF properties for cluster T15. **A)** Mean abundance level of tRFs in the cluster. For every tRF, the relative level is calculated by Z-score normalization. Error bars indicate standard error of the mean. The colors indicate photoperiod (red = LgD; orange = EqD; blue = ShD). tRF number: the number of unique tRF sequences in the cluster. **B)** Pie chart showing the proportion of each tRF class in the cluster by read count (in reads per million, *i.e.,* RPM). Only the top 3 abundant tRF classes by RPM are labeled. **C)** Bar charts showing the total RPM level of each tRF class by fragment size (in number of nucleotides, *i.e.,* nt). Vertical black line separates the shorter tRFs (15–29 nt) on the left from the longer tRFs (30–48 nt) on the right. **D)** Bar charts showing the mapping of tRFs onto the tRNA gene locus for each tRF class. The left, center and right X-axes show the percentile position of the 5′ 100 flaking region, mature tRNA and 3’ flanking region. Mature tRNAs vary in length and therefore the length of each mature tRNA was split into 100 percentiles. The y-axis value indicates the number of tRFs that map to the percentile by BLAST in RPM. The graphs in (B), (C) and (D) are color-coded by the amino acid of the origin tRNA, following the scheme in Fig 3 and Fig 4. Unlabeled tRF classes are in light grey. tRF classes that involve multiple tRNA isotypes are in dark gray.
(EPS)

S25 Fig Summary of tRF properties for cluster T16. **A)** Mean abundance level of tRFs in the cluster. For every tRF, the relative level is calculated by Z-score normalization. Error bars indicate standard error of the mean. The colors indicate photoperiod (red = LgD; orange = EqD; blue = ShD). tRF number: the number of unique tRF sequences in the cluster. **B)** Pie chart showing the proportion of each tRF class in the cluster by read count (in reads per million, *i.e.,* RPM). Only the top 3 abundant tRF classes by RPM are labeled. **C)** Bar charts showing the total RPM level of each tRF class by fragment size (in number of nucleotides, *i.e.,* nt). Vertical black line separates the shorter tRFs (15–29 nt) on the left from the longer tRFs (30–48 nt) on the right. **D)** Bar charts showing the mapping of tRFs onto the tRNA gene locus for each tRF class. The left, center and right X-axes show the percentile position of the 5′ 100 flaking region, mature tRNA and 3’ flanking region. Mature tRNAs vary in length and therefore the length of each mature tRNA was split into 100 percentiles. The y-axis value indicates the number of tRFs that map to the percentile by BLAST in RPM. The graphs in (B), (C) and (D) are color-coded by the amino acid of the origin tRNA, following the scheme in Fig 3 and Fig 4. Unlabeled tRF classes are in light grey. tRF classes that involve multiple tRNA isotypes are in dark gray.
(EPS)

S26 Fig Summary of tRF properties for cluster T17. **A)** Mean abundance level of tRFs in the cluster. For every tRF, the relative level is calculated by Z-score normalization. Error bars indicate standard error of the mean. The colors indicate photoperiod (red = LgD; orange = EqD; blue = ShD). tRF number: the number of unique tRF sequences in the cluster. **B)** Pie chart showing the proportion of each tRF class in the cluster by read count (in reads per million, *i.e.,* RPM). Only the top 3 abundant tRF classes by RPM are labeled. **C)** Bar charts showing the total RPM level of each tRF class by fragment size (in number of nucleotides, *i.e.,* nt). Vertical black line separates the shorter tRFs (15–29 nt) on the left from the longer tRFs (30–48 nt) on the right. **D)** Bar charts showing the mapping of tRFs onto the tRNA gene locus for each tRF class. The left, center and right X-axes show the percentile position of the 5′ 100 flaking region, mature tRNA and 3’ flanking region. Mature tRNAs vary in length and therefore the length of each mature tRNA was split into 100 percentiles. The y-axis value indicates the number of tRFs that map to the percentile by BLAST in RPM. The graphs in (B), (C) and (D) are color-coded by the amino acid of the origin tRNA, following the scheme in Fig 3 and Fig 4. Unlabeled tRF classes are in light grey. tRF classes that involve multiple tRNA isotypes are in dark gray.
(EPS)

S27 Fig Summary of tRF properties for cluster T18. **A)** Mean abundance level of tRFs in the cluster. For every tRF, the relative level is calculated by Z-score normalization. Error bars indicate standard error of the mean. The colors indicate photoperiod (red = LgD; orange = EqD; blue = ShD). tRF number: the number of unique tRF sequences in the cluster. **B)** Pie chart showing the proportion of each tRF class in the cluster by read count (in reads per million, *i.e.,* RPM). Only the top 3 abundant tRF classes by RPM are labeled. **C)** Bar charts showing the total RPM level of each tRF class by fragment size (in number of nucleotides, *i.e.,* nt). Vertical black line separates the shorter tRFs (15–29 nt) on the left from the longer tRFs (30–48 nt) on the right. **D)** Bar charts showing the mapping of tRFs onto the tRNA gene locus for each tRF class. The left, center and right X-axes show the percentile position of the 5′ 100 flaking region, mature tRNA and 3’ flanking region. Mature tRNAs vary in length and therefore the length of each mature tRNA was split into 100 percentiles. The y-axis value indicates the number of tRFs that map to the percentile by BLAST in RPM. The graphs in (B), (C) and (D) are color-coded by the amino acid of the origin tRNA, following the scheme in Fig 3 and Fig 4. Unlabeled tRF classes are in light grey. tRF classes that involve multiple tRNA isotypes are in dark gray.
(EPS)

S28 Fig Summary of tRF properties for cluster T19. **A)** Mean abundance level of tRFs in the cluster. For every tRF, the relative level is calculated by Z-score normalization. Error bars indicate standard error of the mean. The colors indicate photoperiod (red = LgD; orange = EqD; blue = ShD). tRF number: the number of unique tRF sequences in the cluster. **B)** Pie chart showing the proportion of each tRF class in the cluster by read count (in reads per million, *i.e.,* RPM). Only the top 3 abundant tRF classes by RPM are labeled. **C)** Bar charts showing the total RPM level of each tRF class by fragment size (in number of nucleotides, *i.e.,* nt). Vertical black line separates the shorter tRFs (15–29 nt) on the left from the longer tRFs (30–48 nt) on the right. **D)** Bar charts showing the mapping of tRFs onto the tRNA gene locus for each tRF class. The left, center and right X-axes show the percentile position of the 5′ 100 flaking region, mature tRNA and 3’ flanking region. Mature tRNAs vary in length and therefore the length of each mature tRNA was split into 100 percentiles. The y-axis value indicates the number of tRFs that map to the percentile by BLAST in RPM. The graphs in (B), (C) and (D) are color-coded by the amino acid of the origin tRNA, following the scheme in Fig 3 and Fig 4. Unlabeled tRF classes are in light grey. tRF classes that involve multiple tRNA isotypes are in dark gray.
(EPS)

S29 Fig Summary of tRF properties for cluster T20. **A)** Mean abundance level of tRFs in the cluster. For every tRF, the relative level is calculated by Z-score normalization. Error bars indicate standard error of the mean. The colors indicate photoperiod (red = LgD; orange = EqD; blue = ShD). tRF number: the number of unique tRF sequences in the cluster. **B)** Pie chart showing the proportion of each tRF class in the cluster by read count (in reads per million, *i.e.,* RPM). Only the top 3 abundant tRF classes by RPM are labeled. **C)** Bar charts showing the total RPM level of each tRF class by fragment size (in number of nucleotides, *i.e.,* nt). Vertical black line separates the shorter tRFs (15–29 nt) on the left from the longer tRFs (30–48 nt) on the right. **D)** Bar charts showing the mapping of tRFs onto the tRNA gene locus for each tRF class. The left, center and right X-axes show the percentile position of the 5′ 100 flaking region, mature tRNA and 3’ flanking region. Mature tRNAs vary in length and therefore the length of each mature tRNA was split into 100 percentiles. The y-axis value indicates the number of tRFs that map to the percentile by BLAST in RPM. The graphs in (B), (C) and (D) are color-coded by the amino acid of the origin tRNA, following the scheme in Fig 3 and Fig 4. Unlabeled tRF classes are in light grey. tRF classes that involve multiple tRNA isotypes are in dark gray.
(EPS)

S30 Fig Expression levels of trex516 and *CYP71A13* (AT2G30770). Lines indicate mean and colors indicate photoperiod (red = LgD; orange = EqD; blue = ShD). Data points are represented in squares, triangles or circles; the shapes represent the pairing of total RNA samples between s/mRNA-co-sequencing. Asterisk(s) (*) indicates statistical significance of the test of Pearson’s correlation coefficient (PCC). Single (*) and double (**) asterisks indicate adjusted-*p* < 0.05 and ** adjusted-*p* < 0.01, respectively. Expressions are in regularized-log-transformed values (RLD).
(EPS)

S31 Fig Summary statistics for the number of detected photoperiodic transposable elements. **A)** The number of transposable elements (TEs) when quantified at a per-copy level. Light grey: TE copies below expression filter threshold; dark grey: number of TE copies that are < 50% in length to the matched TE copy (considered truncated) or > 10% divergence (considered highly divergent); black: TE copies that are not matched to any TE in the TE library; blue: non photoperiodic TE copies; red: photoperiodic TE copies; **B)** the number of TEs when quantified at a per-family level. The family is presented if at least one copy belonging to the family satisfy the criteria in **(A)**; **C)** bar chart showing the number of aligned (and not aligned) TE copies by RepeatMasker on the RepBase TE library, using the super-family annotation from TAIR; **D)** density plot showing the length distribution of TE copies that were aligned (or not aligned) by RepeatMasker.
(EPS)

S32 Fig Heat map of the clustering of photoperiodic transposable elements at (A) per-copy level and (B) per-family level. **A)** main body: heat map shows the clustering of 65 photoperiodic TE copies into six groups (TEC1 - TEC6; likelihood ratio test: adjusted-*p* < 0.01). **B)** main body: heat map shows the clustering of 38 photoperiodic TE families into four groups (TEF1 - TEF4; likelihood ratio test: adjusted-*p* < 0.01). **For both heatmaps:** photoperiodic DEI Proportion: stacked bar chart of the proportional daily expression integral (DEI) of each gene, raised to the power of twelve for visualization (red = LgD; orange = EqD; blue = ShD); total expression: heat map of the total expression in RLD-normalized read counts.
(EPS)

S33 Fig Quantile-quantile plot of the *p*-values for Pearson’s correlation test for tRF-TE pairs. The observed unadjusted-*p*-value are plotted against the expected *p*-value for the Pearson’s correlation test for each miRNA-mRNA pair in an analysis with (A) LgD samples only, (B) EqD samples only, (C) ShD samples only and (D) across all samples in LgD, EqD and ShD. Axis are in -log_10_ scale. Straight line indicates the values where observed *p*-values equal to expected *p*-values. Red and blue dots indicate positively and negatively correlated pairs, respectively.
(EPS)

S34 Fig Violin plot of the Pearson’s correlation coefficient for tRF-TE pairs. Distributions of Pearson’s correlation coefficients (PCCs) of tRF-TE pairs calculated using LgD samples only, EqD samples only, ShD samples only, and all samples are represented by the gray, red, orange and blue violins. Individual dots indicate individual pairs.
(EPS)

S35 FigExpressions of selected genes involved in sRNA processing.Dots indicate individual biological samples. Lines indicate mean. The colors of dots and lines indicate photoperiod (red = LgD; orange = EqD; blue = ShD). *DICER-LIKE 1* (*DCL1*), *HYPONASTIC LEAVES 1* (*HYL1*), *ARGONAUTE 1* (*AGO1*), *MRNA ADENOSINE METHYLASE* (*MTA*), *HUA ENHANCER 1* (*HEN1*), *CYCLIN-DEPENDENT KINASE F;1* (*CDKF;1*) and *REDUCTION IN BLEACHED VEIN AREA* (*RBV*) are photoperiodic with adjusted-*p* < 0.01. *SERRATE* (*SE*) is not photoperiodic (adjusted-*p* ≈ 0.0124).(EPS)

S36 FigExpressions of *RNS1*/*2*/*3* (*AT2G02990*/*AT2G39780*/*AT1G26820*).**Dots indicate individual biological samples.** Lines indicate mean. The colors of dots and lines indicate photoperiod (red = LgD; orange = EqD; blue = ShD). Both *RNS1*/*2* are photoperiodically regulated (adjusted-*p* < 0.01), while *RNS3* is not (adjusted-*p* ≈ 0.0488).(EPS)

S37 Fig Expressions of reported HY5-regulated miRNAs [44], including miR172ab, miR1888ab, miR402, miR408-5/3p, miR858ab and miR869.1/2. Dots indicate individual biological samples. Lines indicate mean. The colors of dots and lines indicate photoperiod (red = LgD; orange = EqD; blue = ShD).
(EPS)

S38 Fig Expressions of *ATHILA6A* TE family and trex8859. Dots indicate individual biological samples. Lines indicate mean. The colors of dots and lines indicate photoperiod (red = LgD; orange = EqD; blue = ShD).
(EPS)

S1 Data Descriptive statistics of the sRNA-sequencing libraries and the mapping process in Excel spreadsheets. (XLSX)

S2 Data Read counts of miRNA in comma delimited format. (CSV)

S3 Data Results of the likelihood ratio test implemented by DESeq2 and clustering results for miRNAs in Excel spreadsheets. (XLSX)

S4 Data Salmon-quantified pseudo-counts of the mRNA-sequencing re-analysis in comma delimited format. (CSV)

S5 Data Results of the likelihood ratio test implemented by DESeq2 and clustering results for mRNAs and transposable elements (including annotations) in Excel spreadsheets. (XLSX)

S6 Data Summary of analyses on the photoperiodic miRNA-mRNA pairs in Excel spreadsheets. (XLSX)

S7 Data Summary of analyses on the time-shifted photoperiodic miRNA-mRNA pairs in Excel spreadsheets. (XLSX)

S8 Data Read counts of tRFs in comma delimited format. (ZIP)

S9 Data Results of the likelihood ratio test implemented by DESeq2 and clustering results for tRFs in Excel spreadsheets. (XLSX)

S10 Data RepeatMasker results generated by calcDivergenceFromAlign.pl. This contains the alignment of TAIR TE annotations to TE library. This also contains the list of TE copies and TE families that were matched to the TE library.
(XLSX)

S11 Data Read counts of transposable elements from the mRNA-sequencing re-analysis in Excel spreadsheets. This contains the results of TElocal and TEcount, for per-copy level and per-family level quantifications, respectively.
(XLSX)

S12 Data Summary of psRNATarget results for tRF target predictions. (XLSX)

S13 Data Summary of analyses on the photoperiodic tRF-TE pairs, at both per-copy and per-family levels, in Excel spreadsheets. (XLSX)
